# Biochemical and antidiabetic properties of *Elaeocarpus angustifolius* Blume: *In vitro, In vivo,* and *In silico* insights

**DOI:** 10.1371/journal.pone.0349796

**Published:** 2026-06-08

**Authors:** Sabina Panth, Rojina Devkota, Amar Waiba, Tika Ram Lamichhane, Prajwal Acharya, Begum Rokeya, Amit Jaisi, Achyut Adhikari

**Affiliations:** 1 Central Department of Chemistry, Tribhuvan University, Kathmandu, Nepal; 2 Central Department of Physics, Tribhuvan University, Kathmandu, Nepal; 3 Department of Pharmacology, Bangladesh University of Health Sciences, Dhaka, Bangladesh; 4 Department of Chemistry, Faculty of Science, Chulalongkorn University, Bangkok, Thailand; Universidade Federal do Para, BRAZIL

## Abstract

Diabetes poses an utmost threat to human health; significant progress in the discovery of antidiabetic drugs has been made, but their side effects cannot be ignored. As a plant-based alternative, this study explored the pharmacological potential of *Elaeocarpus angustifolius* Blume (Rudraksha), emphasizing its antioxidant and antidiabetic activities, supported by *in silico* analysis. Phytochemical screening of the methanol bark extract confirmed the presence of flavonoids, alkaloids, carbohydrates, glycosides, terpenoids, tannins, and phenols. The extract exhibited high total phenolic and flavonoid contents (182.73 ± 0.001 mg GAE/g and 48.78 ± 0.06 mg QE/g, respectively). The LC–MS analysis of the ethyl acetate fraction identified thirteen phytoconstituents, including phenolic acids, flavonoids, and tannins. The ethyl acetate fraction demonstrated significant antioxidant activity (IC₅₀ = 1.47 ± 0.37 µg/mL), outperforming quercetin (IC₅₀ = 4.87 ± 0.16 µg/mL). Enzyme inhibition assays revealed methanol extract has strong α-glucosidase (IC₅₀ = 0.79 ± 0.13 µg/mL) and α-amylase (IC₅₀ = 3.48 ± 0.00 µg/mL) inhibitory effects, comparable to acarbose (IC₅₀ = 13.51 ± 0.22 µg/mL and IC₅₀ = 25.30 ± 0.85 µg/mL), respectively. In a 28-day *in vivo* study, oral administration of the methanol bark extract significantly (p < 0.01) reduced fasting serum glucose levels and improved lipid profiles, with decreases in triglycerides (5%) and total cholesterol (2%), and an increase in HDL (5%) compared with baseline. The extract-treated group also showed higher hepatic glycogen content (14.06 ± 0.002 mg/mL) than the Gliclazide-treated group (12.82 ± 0.002 mg/mL). *In silico* molecular docking identified Corilagin exhibiting the highest binding affinity (−10.3 kcal/mol), stable interaction (RMSD = 1.469 ± 0.133 Å), and favorable binding free energy (ΔG_BFE_ = −27.48 ± 3.15) with α-amylase, suggesting that it may act as a competitive inhibitor of an enzyme’s active site. Overall, these findings highlight that *E. angustifolius* bark significantly inhibits glucose absorption and improves dyslipidemia to some extent, while *in silico* analysis suggests that Corilagin has potential as an inhibitory activity against type-2 diabetes.

## 1. Introduction

Diabetes mellitus is the most common endocrine disorder, resulting from insufficient insulin production or the body’s inability to use insulin effectively, ensuing in abnormal glucose concentrations in the blood [1]. The prevalence of diabetes in urban areas has dramatically increased across numerous Asian countries, with a substantial rise seen in places like Nepal, Bangladesh, India, Bhutan, Sri Lanka, Pakistan, and China. etc [2]. Over the past few decades, there has been a consistent increase in the number of people who have diabetes. The International Diabetes Federation Atlas, 11^th^ edition, predicted that 589 million adults were living with diabetes. Also, the global population of individuals living with diabetes is anticipated to grow to 853 million by 2050 [3]. Diabetes mellitus (DM) presents an elevated risk of numerous consequences, including heart disease, peripheral vascular conditions, stroke, nerve damage, kidney failure, vision loss due to retinopathy, etc [4].

People with type 2 diabetes mellitus are more likely to experience a variety of immediate and long-term problems, which typically contribute to premature mortality [5]. Diabetes can be treated with a wide range of hypoglycemic agents or medications, including biguanides, sulfonylureas, and metformin [6]. However, none of these treatments are optimal because of their harmful side effects, and responses are occasionally reduced, sometimes with prolonged use. A fundamental drawback of these treatments is that they require lifelong prescriptions and often lead to undesirable side effects [7]. The use of therapeutic herbs that possess hypoglycemic properties has gained popularity in recent years [8]. The presence of carotenoids, flavonoids, polysaccharides, terpenoids, alkaloids, and glycosides in most plants exhibits antidiabetic properties [9]. The antiglycemic effect of plant therapy is ascribed to the plant’s ability to suppress pancreatic tissue function, which is accomplished by either boosting insulin production or reducing intestinal glucose absorption [10]*.*

*Elaeocarpus angustifolius*, commonly referred as Rudraksha, belongs to the *Elaeocarpaceae* family, which is about 50−200 feet long and possesses a cylindrical trunk with greyish white and rough-textured bark. Its captivating fruit stones and therapeutic medicinal properties make it popular [11]. Traditionally, Rudraksha’s bark, beads, and leaves are used to treat a wide range of conditions, such as diabetes, anxiety, depression, nerve pain, epilepsy, migraine, stress, microbial infections, etc [12]. In the context of Nepal, people from remote areas believe this plant is useful for managing diabetes. In general, *Elaeocarpus* species are characterized by their presence of numerous compounds, including triterpenes, tannins, saponins, alkaloids, flavonoids, terpenoids, etc. [13]. This abundance of bioactive compounds makes Rudraksha an effective herbal remedy for addressing a variety of ailments [13]. Several scientific reports demonstrated that different fractions of bark, leaves, or fruit significantly reduce glucose levels and are used to treat type-2 diabetes. However, studies on phytochemical compounds of *Elaeocarpus* species and their antidiabetic activities with *in silico* insights have been limited. Hence, this study aims to explore antioxidants*, in vivo* and *in vitro* antidiabetic activities of the *Elaeocarpus angustifolius* blume. Nonetheless, animal models remain an invaluable tool for understanding the biological response and disease related complications, as well as translating experimental findings to medical applications. Additionally, this research plans to conduct *in silico* molecular docking, molecular dynamics, free energy, and assess ADMET toxicity parameters to support our *in vitro* and *in vivo* findings and elucidate the mechanism responsible for biological activity.

## 2. Materials and methods

### 2.1 Chemicals

Methanol (CH_3_OH), Ethanol (CH_3_CH_2_OH), Sodium hydroxide (NaOH), Hexane, Dichloromethane (DCM), Ethylacetate (C_4_H_8_O_2_), Dimethylsulfoxide (DMSO), Sodium carbonate (Na_2_CO_3_), Aluminium trichloride (AlCl_3_), Di-potassium hydrogen orthophosphate, Triple de-ionised water, Potassium acetate (CH_3_COOK), Potassium dihydrogen orthophosphate, were purchased from a local supplier. Quercetin, Folin-Ciocalteu reagent (FCR), Gallic acid, 2, 2-diphenyl-1-picryhydrazyl (DPPH), 2-chloro-4-nitrophenyl-α-maltotrioside (CNPG3), Acarbose, P-nitrophenyl-α-D-glucopyranoside (PNPG), α-glucosidase from Saccharomyces cerevisiae, were purchased from Sigma-Aldrich, Germany. Streptozotocin (STZ) (Sigma Aldrich), Glucose oxidase (GOD-PAP) reagents, Sulphuric acid, Rat Insulin ELISA kit, Cholesterol oxidase/peroxidase (CHOD-PAP) reagent, Enzymatic colourimetric (GPO-PAP) reagent, and Gliclazide were purchased from Randox Laboratories.

### 2.2 Plant materials

The fresh bark of *E. angustifolius* was collected from Kirtipur, Kathmandu, situated at 27°40’56”N and 85°15’15’‘ at about 1400 m above sea level. The plants underwent taxonomical identification at the National Herbarium and Plant Laboratories, Godavari, Lalitpur, Nepal (Voucher code no: 110 KATHI 64207).

### 2.3 Preparation of type-2 diabetic model rats

Long-Evans rats sourced from Bangladesh University of Health Sciences (BUHS) animal house, where healthy adult rats with body weights ranging from 195g to 295g were chosen for this study. The animals were fed with a conventional commercial pellet diet and boiled water. All the rats were kept in the same environment, and they maintained standard conditions of humidity (40−70%), temperature (22 ± 2°C), and light (12-hour light and 12-hour dark cycle). Type-2 diabetes was induced by injecting streptozotocin into citrate buffer (pH 4.5) at a dose of 90 mg/mL into neonatal rat pups within 48 hours of their birth. The development of type-2 diabetes mellitus in the Long-Evans rats was confirmed by testing their blood serum glucose level with the oral glucose tolerance test (OGTT). The experiment was performed based on ARRIVE guidelines. The animals were first ethically approved by the Ethical Review Committee (ERC) of the Bangladesh University of Health Sciences with a Memo No: BUHS/ERC/EA/23/62.

### 2.4 Experimental design

Twenty-four rats (6 normal; 18 STZ-induced diabetic rats) were allocated into four groups, each including six rats. Group 1: Normal water control (NWC) treated (10mL/kg bw), Group 2: Diabetes water control (DWC) (10mL/kg bw), Group 3: Gliclazide treated (GT) (20 mg/kg/5mL), and Group 4: Extract treated (*Elaeocarpus angustifolius* bark) received 1.25g/kg/10 ml. All the rats were fed extracts, water, and a control orally for 28 days. During the experiment, the body weight was recorded five times: on the 0^th^ day, 7^th^ day, 14^th^ day, 21^st^ day, and 28^th^ day. The blood was collected at 0 and 28 days. On the 0^th^ day, blood was drawn from the tail vein, while on the 28^th^ day, blood was collected from the heart via the cardiac puncture method. After 30 minutes of blood collection, it was centrifuged at normal room temperature at 3700 rpm.

### 2.5 Preparation of plant extract

The bark of *E. angustifolius* was collected, cleaned, and allowed to dry at room temperature without exposure to sunlight. They were then finely ground into a powder using a grinding machine. The powdered plants were extracted using methanol through the maceration method [14]. The powdered sample was placed in a clean, dry conical flask, and pure (100%) methanol was added, and then left for three days. The mixture was then filtered out using filter paper; similarly, filtrates were collected in a separate beaker. The residue was further subjected to three additional rounds of extraction using the same process. All the combined filtrates were filtered through Whatman filter paper and were concentrated at 45 ^°^C using a rotary evaporator. Then, the remaining filtrate from each sample was placed in a water bath at 37 ^°^C to obtain a dried sample. The resulting extract was weighed to determine the yield percentage and stored in a refrigerator at 4 ^°^C for further analysis. Also, the methanol extract was dissolved in water, and three successive fractionations were carried out using hexane, DCM, and ethyl acetate.

### 2.6 Phytochemical screening

The methanol extract of *E. angustifolius* bark was subjected to a preliminary qualitative phytochemical screening to detect the plant components. All the phytochemical screening of the extract was carried out in accordance with the protocol described by [15].

### 2.7 Total phenolic content (TPC) and total flavonoid content (TFC)

The Folin-Ciocalteu colourimetric method based on oxidation-reduction reaction process was used to assess total phenolic content in the plant extract [16]. The reaction mixture was prepared by adding 20 µL of plant extract with a concentration of 1 mg/mL, 100 µL of 10% Folin-Ciocalteu (FC) reagent, and 80 µL of 1M sodium carbonate (Na_2_CO_3_). This reaction mixture then allowed to stand at room temperature in dark for 20 minutes. The maximum absorbance was measured at 765 nm. The standard solution of gallic acid undergoes the same procedure. The total phenolic content in the plant extract was determined as milligrams of gallic acid equivalent per gram (mg GAE/g) of plant extract.

Similarly, Total flavonoid content was determined by using the aluminium chloride colourimetric method with slight modifications [17]. The mixture was then prepared by combining 20 µL of plant extract with a concentration of 1 mg/mL, 110 µL of distilled water, and 60 µL of ethanol. After that, 5 µL of AlCl_3_ and 5 µL of CH_3_COOK were added simultaneously. The mixture was incubated at room temperature for 30 minutes. The standard Quercetin solution undergoes the same procedure. The maximum absorbance was taken at 415 nm. The total flavonoid content in the plant extract was determined as milligrams of quercetin equivalent per gram (mg QE/g) of plant extract.

### 2.8 *In vitro* antioxidant activity

The free radical scavenging activity of the extract and fractions was assessed by the DPPH (1, 1 diphenyl-2- picryl- hydrazyl) free radical scavenging method with slight modifications [18]. Standard quercetin and methanol extract and its fractions of varying concentrations (100 µL each) were loaded into separate well plates. Subsequently, 100 µL of (0.1mM) DPPH was added. The mixture was incubated in the dark at room temperature for 30 min. The absorbance was then taken at 517 nm. Finally, % inhibition was then calculated by using the following formula.


$$\begin{array}{c} \%\;inhibition\;=\frac{{\text{A}}_{c}-{\text{A}}_{s}}{{\text{A}}_{c}}\;\times \text{1}\text{0}\text{0}\; \end{array}$$ (1)

Where A_c_ and A_s_ are the absorbance of the control and sample.

### 2.9 *In vitro* α-glucosidase inhibition activity

A modified version of a previously known method was used for the α-glucosidase inhibition assay for the extract and fractions [19]. The mixture of 40 µL of plant extract of various concentrations, along with 10 µL of α-glucosidase (0.20 units/mL) enzyme and 80 µL of phosphate buffer solution, was pre-incubated at 37 ^°^C for 10 minutes. Subsequently, 20 µL of *pNPG* substrate and 50 µL of Na_2_CO_3_ were added to the mixture. The yellow colour produced by *p-*nitrophenol was measured at 405 nm. The same process was used to the standard Acarbose. % inhibition was then calculated by using the formula as mentioned in equation 1.

### 2.10 *In vitro* α-amylase inhibition activity

The α-amylase inhibition assay for the extract and fractions followed a modified version of a previously established method [20]. The mixture of 20 µL of plant extract of various concentrations, along with 80 µL of α- amylase (1.5 units/mL) enzyme, was pre-incubated at 37 ^°^C for 10 minutes. Subsequently, 100 µL of *CNPG3* substrate was added to the mixture. The yellow color produced by chloro-nitrophenol was measured at 405 nm. The standard Acarbose underwent the same procedures. % inhibition was then calculated by using the formula as shown in equation 1.

### 2.11 LC-MS analysis

An LC-ESI/MS system was used to examine the 100% ethylacetate extract of *E. angustifolius* bark. Liquid chromatography was conducted using Thermo Dionex Ultimate 3000 coupled with a SCIEX Triple TOF ^®^ 6600 + . The mobile phase consisted of 0.1% formic acid in water (A) and 0.1% formic acid in acetonitrile (B), flowing at 0.3mL/min in a linear gradient as follows: (Time. % B): 0 min-5, 2 min −5, 20 min- 95, 25 min- 95, 25.5 min- 5, 30 min – 5. Analysis was done by using 2 µL of plant extract at a concentration of 1 mg/mL. An integrated single quadrupole mass analyzer was used to perform mass spectrometric detection using ESI in both positive and negative modes, scanning from m/z 100–1500 Da. The analysis was conducted using SCIEX OS software version 3.3.0.12027, with library searches performed against NIST 2017 and the natural product HR-MS/MS 2.0 database.

### 2.12 *In vivo* assay

#### 2.12.1 Serum glucose level.

The glucose Oxidase (GOD-PAP) method was used for the estimation of serum glucose level. Blood collected for 0 days and 28 days was then centrifuged for 4–10 minutes (3500–4000 rpm), and serum separation was done. 5 µL of sample and 250 µL of GOD-PAP reagent were combined and then incubated at 37 °C for 15 minutes. The absorbance was taken at 490–510 WL. The standard glucose (20Mm) of varying concentrations undergoes the same procedure.

#### 2.12.2 Serum cholesterol measurement.

Cholesterol level was determined using the colorimetric (CHOD-PAP) method [21]. 5 µL of sample serum and 200 µL of CHOD-PAP reagent were combined, and incubation at 37 °C for 5 minutes. The absorbance was taken at 490–510 nm.

#### 2.12.3 Serum triglyceride measurement.

The enzymatic colourimetric (GPO-PAP) method was applied for the determination of serum TG [22]. 5 µL serum sample and 200 µL of GPO-PAP reagent were combined, and incubation was done for 5 minutes at 37 °C. The absorbance was taken at 490–510 WL.

#### 2.12.4 Serum high-density lipoprotein (HDL).

The colourimetric (CHOD-PAP) method was used to determine the high-density lipoprotein (HDL). Sample serum and precipitant were mixed (1:2 ratios) and centrifuged for 10 minutes (3500–4000 rpm). 20 µL supernatant sample and 200µLof CHOD-PAP reagent were mixed, followed by incubation for at 37 °C for 5 minutes. The absorbance was taken at 490–510 WL.

#### 2.12.5 Low-density lipoprotein (LDL).

LDL was estimated by using the following formula:


$$\begin{array}{c} LDL=(total\;cholesterol-HDL)-(TG/\text{5}) \end{array}$$ (2)

#### 2.12.6 Glycogen level.

Measurement of glycogen from rat liver was done by the Anthrone Sulphuric acid method [23]. 200 mg of rat liver was taken, and then 5% of TCA (Trichloroacetic acid) was used to homogenize the liver and the suspension was then filtered to obtain a watery-like solution as a sample. After that, 2mL of KOH (10N) and 1mL of TCA were added to each of the sample solutions, like DWC, NWC, GT, and extract one. Similarly, after 2–3 minutes of shaking, incubation for 1 hour in paraffin solution at 100 °C was performed. After incubation, 1mL of glacial acetic acid and 2 mL of anthrone solution were added. Again, the solution is incubated in paraffin solution at 100 °C for 10 min. After incubation sample solution is cooled, and 200 µL of the sample solution is taken to observe the absorbance. The absorbance was taken with a 650 nm – 492nm range. The same procedure is done for standard glucose solutions.

### 2.13 Statistical analysis

The *in vitro* statistical analysis was done with GraphPad Prism version 8.0 for Windows. Values are expressed as the mean ± SD. Statistical evaluation of *in vivo* data was performed by using Statistical Package for Social Science (SPSS) for windows 10. Data has been expressed as mean ± SD. One-way analysis of variance (ANOVA) and a paired sample t-test was used to perform statistical comparison between groups. The significance level was considered at p < 0.05.

### 2.14 Computational method

#### 2.14.1 Molecular docking studies.

**Fig 1:**
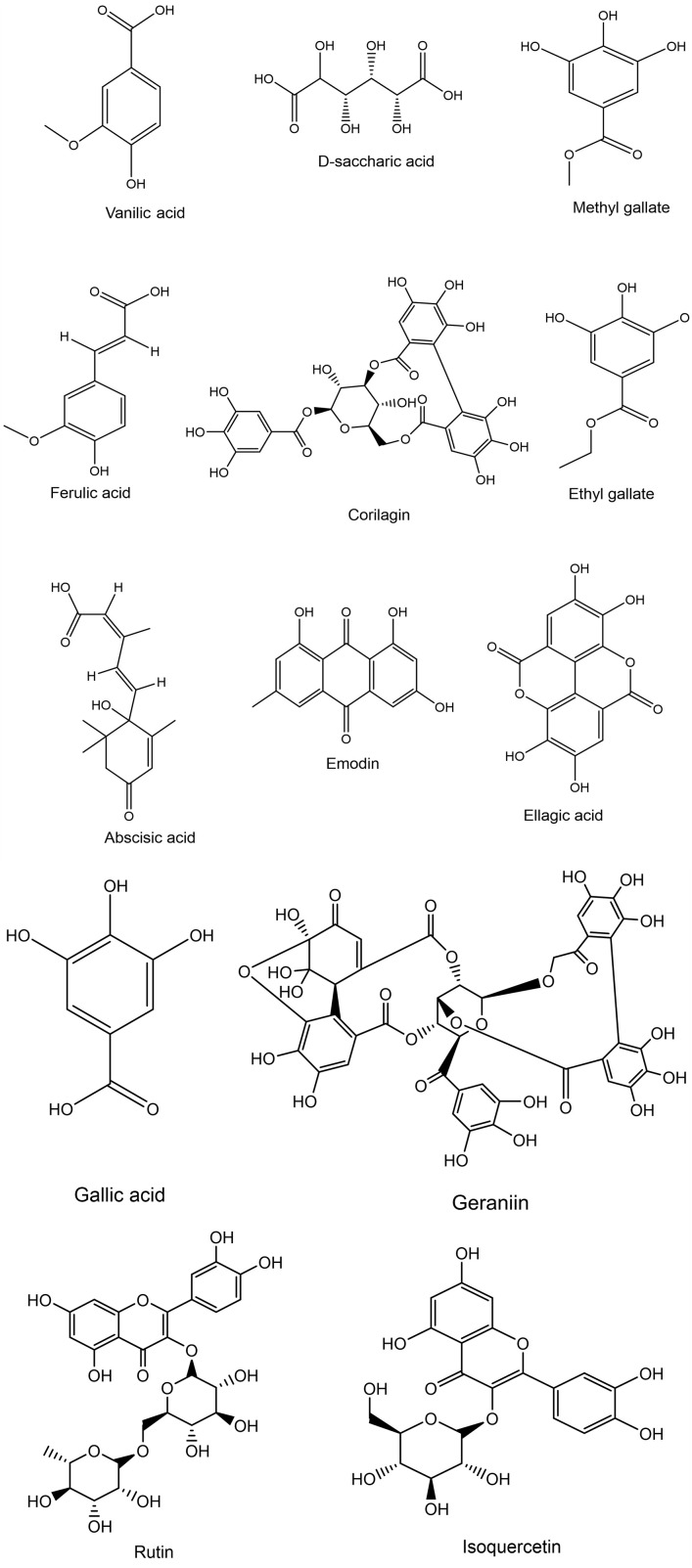
Chemical structures of phytoconstituents identified from the ethylacetate fraction of *E. angustifolius.*

The structure of identified ligands was retrieved from the PubChem server in the form of SDF, which was optimized and then converted into PDB by using Avogadro’s software (Fig 1) [24,25]. The Protein Data Bank server (https://www.rcsb.org/) was utilized to obtain proteins α-glucosidase (PDB ID: 3A4A) and α-amylase (PDB ID: 2QV4) in PDB format, with resolution was 1.60 Å and 1.97 Å, respectively [26,27]. First, the proteins were cleaned by removing water molecules, co-crystallized ligand, and adding hydrogen bonds by using Biovia Discovery Studio Visualizer to prepare them for further analysis. Afterwards, AutoDock Vina v1.5.7, which adds a kollman charges of −30.6 and 7.376 for alpha-glucosidase and alpha amylase, respectively, to make the system neutral. Using AutoDock Tools v1.5.7, the protein and ligand structures were converted from PDB to PDBQT format and AutoDock Vina was used to conduct molecular docking [28,29]. The box size for alpha-glucosidase and alpha amylase was 40, 40, 40 grid points with spacing 0.375 Å with an energy range of 4 and 20 modes, with an exhaustiveness of 32. The grid centers were set for (X = 23.244385, Y = −7.482308, Z = 23.570923) and (X = 12.4367, Y = 48.132, Z = 26.1819) for alpha-glucosidase and alpha amylase, respectively. The CASTp server and various literature sources were used to confirm the orthosteric sites of the proteins [26,30]. The interaction of the protein and ligands was investigated using Biovia Discovery Studio visualizer software [31]. The docking protocol was validated by achieving an RMSD of less than 2 Å between heavy atoms in the docked pose and the crystal structure (S1 Fig).

#### 2.14.2 Molecular dynamics simulation.

The MD simulations were carried out using GROMACS v2019.6 [32]. For the receptor topology, the CHARMM27 force field was used due to its compatibility with ligand topology files obtained from the Swiss Param server [33,34]. The MD simulations of the best binding protein-ligand complex were conducted out with a time-step of 2 fs per step in TIP3P water as the solvent in a dodecahedron box neutralized with Na^+^ and Cl^-^ ions at a constant temperature of 300 K. The NVT and NPT runs were each 100 ps long, and the output was run for 100 ns long, with a time interval of 100 ps between frames to generate 1000 frames MD trajectories. These were descriptions of the typical procedures involved in running an MD simulation. To determine the protein-ligand complex’s stability, changes in conformation, and binding effectiveness, parameters like Root Mean Square Deviation (RMSD), Root Mean Square Fluctuation (RMSF), Radius of Gyration (Rg), and Solvent Accessible Surface Area (SASA) were obtained by using GROMACS’ inbuilt modules, while H-bond count was obtained by using VMD v1.9.3 with donor acceptor distance of 3.5 Å [35].

#### 2.14.3 Binding free energy calculations.

The molecular mechanics Poisson-Boltzmann surface area (MM/PBSA) method was adopted to calculate the variations in the binding free energies of the protein-ligand complex using equation 3 [36].


$$\begin{array}{c} {\Delta \text{G}}_{\text{BFE}}={\Delta \text{G}}_{\text{complex}}-({\Delta \text{G}}_{\text{protein}}+\;{\Delta \text{G}}_{\text{ligand}}) \end{array}$$ (3)

$$\begin{array}{c} {\Delta \text{G}}_{\text{BFE}}={\Delta \text{G}}_{\text{gas}}+{\Delta \text{G}}_{\text{solv}} \end{array}$$ (4)

ΔG_gas_ is the total of electrostatic (ΔE_EL_) and van der Waals energies (ΔE_VDW_), whereas ΔG_solv_ is the sum of polar (ΔE_PB_) and nonpolar (ΔE_NPOLAR_) components [37].


$$\begin{array}{c} {\Delta \text{G}}_{\text{BFE}}={\Delta \text{G}}_{\text{VDW}}+{\Delta \text{G}}_{\text{EL}}+{\Delta \text{G}}_{\text{PB}}+{\Delta \text{G}}_{\text{NPOLAR}} \end{array}$$ (5)

The spontaneity and viability of the forward reaction were evaluated based on the sign of free energy changes. Here, 100 frames representing 10 ns of the equilibrated final part of the MDS trajectory were used.

#### 2.14.4 ADMETanalysis.

For evaluating the pharmacokinetic and pharmacodynamic properties of compounds were evaluated from ProTox-III, pkCSM, ADMETlab2.0, and Swiss ADME web servers [38–41].

## 3. Results and discussion

### 3.1 Phytochemical screening

The total yield percentage obtained from the methanol extract of *E. angustifolius* bark is 12.40%. The methanol extract of *E. angustifolius* bark showed the presence of phytochemical constituents such as tannins, phenols, alkaloids, terpenoids, flavonoids, reducing sugar, saponins, volatiles, carbohydrates, and glycosides (S1 Table). These results demonstrate the abundance of various phytochemicals in the extracts, underscoring their potential medicinal value. Similarly, a previous study on the phytochemical screening of different parts of the *Elaeocarpus* plants revealed that phenolic, terpenoids, saponin, and flavonoids are the most prevalent phytochemicals present in it, as compared to other phytoconstituents, which are comparable to this work [42].

### 3.2 Total phenolic content

The total phenolic content of the methanol extract of *E. angustifolius* bark was assessed through folin-ciocalteu assay, employing gallic acid as the standard that can be quantified at 765 nm using a visible light spectrophotometer. A calibration curve was created by plotting gallic acid absorption versus concentration (S2 Fig). The total phenolic content was determined from this curve using the regression equation Y = 0.0072x + 0.01 and R^2^ = 0.9988 followed by C = cV/M and expressed as mg gallic acid equivalent (GAE) per gram of extract in dry weight. The phenolic content of the methanol extract of bark was found to be 182.73 ± 0.001 mg GAE/g, respectively, in dry weight. Similarly, the TPC and TFC values obtained from this study are comparable to the results reported by previous findings on *E. ganitrus* seeds [43]. The quantitative estimation of total phenolic content showed that the extract is rich in phenolic compounds, which possess physiological activity.

### 3.3 Total flavonoid content

The total flavonoid content of the methanol extract of *E. angustifolius* bark was determined using a colorimetric assay, aluminium chloride method, where quercetin was used as a standard reference for the determination of the calibration curve. The absorption was taken at 415 nm using a UV-VS spectrophotometer with different concentrations, and then the graph was plotted as absorption versus concentration (S3 Fig). The regression equation Y = 0.0232x + 0.0028 and R^2^ = 0.9907 followed by C = cV/M was used to determine the total flavonoid content and expressed as mg quercetin equivalent per gram (mg QE/g) from the calibration curve. The flavonoid content of the methanol extract of bark was found to be 48.78 ± 0.06 mg QE/g, respectively, in dry weights. The total flavonoid content of the *E. angustifolius* bark showed bark was rich in flavonoids, which might exhibit a wide range of medicinal properties. Also, the TPC and TFC values obtained from this study showed higher values in comparison to the values obtained from *E. sphaericus* methanolic leaf extract [44]. The presence of these secondary metabolites in the plant extract helps in reducing various biological ailments.

### 3.4 *In vitro* antioxidant activity

The antioxidant activity of the methanol extract and fractions of *E. angustifolius* bark was determined by the DPPH free radical scavenging method. The antioxidant activity of *E. angustifolius* bark and its fractions (hexane, DCM, ethylacetate, aqueous) is calculated (S2 Table). The antioxidant activity of the crude extract of *E. angustifolius* bark was found to be 1.48 ± 0.39 µg/mL. Similarly, ethylacetate fractions of *E. angustifolius* bark showed the minimum IC_50_ value of 1.47 ± 0.37 µg/mL, while the DCM fractions showed the maximum IC_50_ value of 13.46 ± 0.00 µg/mL. Additionally, Hexane fractions showed the IC_50_ value of 6.48 ± 1.11 µg/mL, and aqueous fractions with an IC_50_ value of 1.48 ± 0.28 µg/mL. The DPPH free radical scavenging activity of *E. angustifolius* crude and fractions shows comparable activity with the standard quercetin (4.87 ± 0.16 µg/mL). The previously reported antioxidant activity of the methanolic extract of *E. floribundus* bark and the ethyl acetate extract of leaves exhibited an IC_50_ value of 7.36 ± 0.01 and 9.37 ± 0.06 µg/mL, respectively, which is significantly comparable to this work [45]. Therefore, the bark of *E. angustifolius* possesses superior antioxidant activity due to the presence of flavonoids, phenols, and other classes of bioactive secondary metabolites.

### 3.5 *In vitro* α-glucosidase activity

The antidiabetic activity of the plant extract was assessed by α-glucosidase inhibitory assay, where acarbose is used as a standard. Crude extracts of *E. angustifolius* bark, along with their fractions, were tested *in vitro,* and the data were analysed based on their IC_50_ values (S3 Table). The IC_50_ value of crude methanol extracts of *E. angustifolius* bark was found to be 0.79 ± 0.13 µg/mL. Similarly, among the fractions of *E. angustifolius* bark, aqueous fractions showed the maximum α-Glucosidase inhibition activity with an IC_50_ value of 1.46 ± 0.30 µg/mL. The DCM fraction showed the least inhibition activity with an IC_50_ value of 16.97 ± 0.22 µg/mL. Likewise, ethyl acetate fractions showed an IC_50_ value of 1.79 ± 0.29 µg/mL, and hexane fractions showed an IC_50_ value of 1.73 ± 0.02 µg/mL, respectively. The extract and fractions show strong considerable inhibitory efficacy against α-glucosidase in comparison to standard acarbose 13.51 ± 0.22 µg/mL. Likewise, previous studies demonstrated comparable inhibition activity of the n-butanol fraction of *Elaeocarpus* bark against α-glucosidase with an IC_50_ value of 2.32 ± 0.62 µg/mL [46].

### 3.6 *In vitro* α-amylase activity

The results showed that the methanol extract exhibited high enzyme inhibition capacity against α-amylase with an IC_50_ value of 3.48 ± 0.00 µg/mL. Similarly, the aqueous fraction shows 4.79 ± 0.06 µg/mL, whereas the DCM fraction showed a considerably higher IC_50_ value of 23.53 ± 0.18 µg/mL. Likewise, hexane and ethyl acetate fractions demonstrated IC_50_ values of 10.33 ± 0.15 µg/mL and 9.36 ± 0.38 µg/mL, respectively. These were more active than the standard acarbose with an IC_50_ value of 25.30 ± 0.85 µg/mL (S4 Table). Similarly, enzyme inhibition activity in bark methanol extract was observed in a previous report against α-amylase with an IC_50_ value of 2.81 ± 0.37 µg/mL [46].

### 3.7 *In vivo* assay

#### 3.7.1 Body weight of animals.

During the 28^th^ day experimental period, the body weight of each rat was measured at every 7^th^ day interval. The body weight of NWC, DWC, and EA (*E. angustifolius*) treated groups increased gradually by 10%, 4%, and 22%, respectively, compared to their 0^th^ day body weight. But the body weight of the GT-treated groups was slightly decreased by 2% compared to their 0^th^ day body weight. However, compared with NWC, DWC, GT, and EA treated groups, NWC vs. EA has shown a significant reduction (p < 0.001), DWC vs. EA has shown a significant reduction (p < 0.001), and GT vs. EA has shown a significant reduction (p < 0.001) of body weight on the final day, respectively (S5 Table).

#### 3.7.2 Serum glucose level.

**Fig 2:**
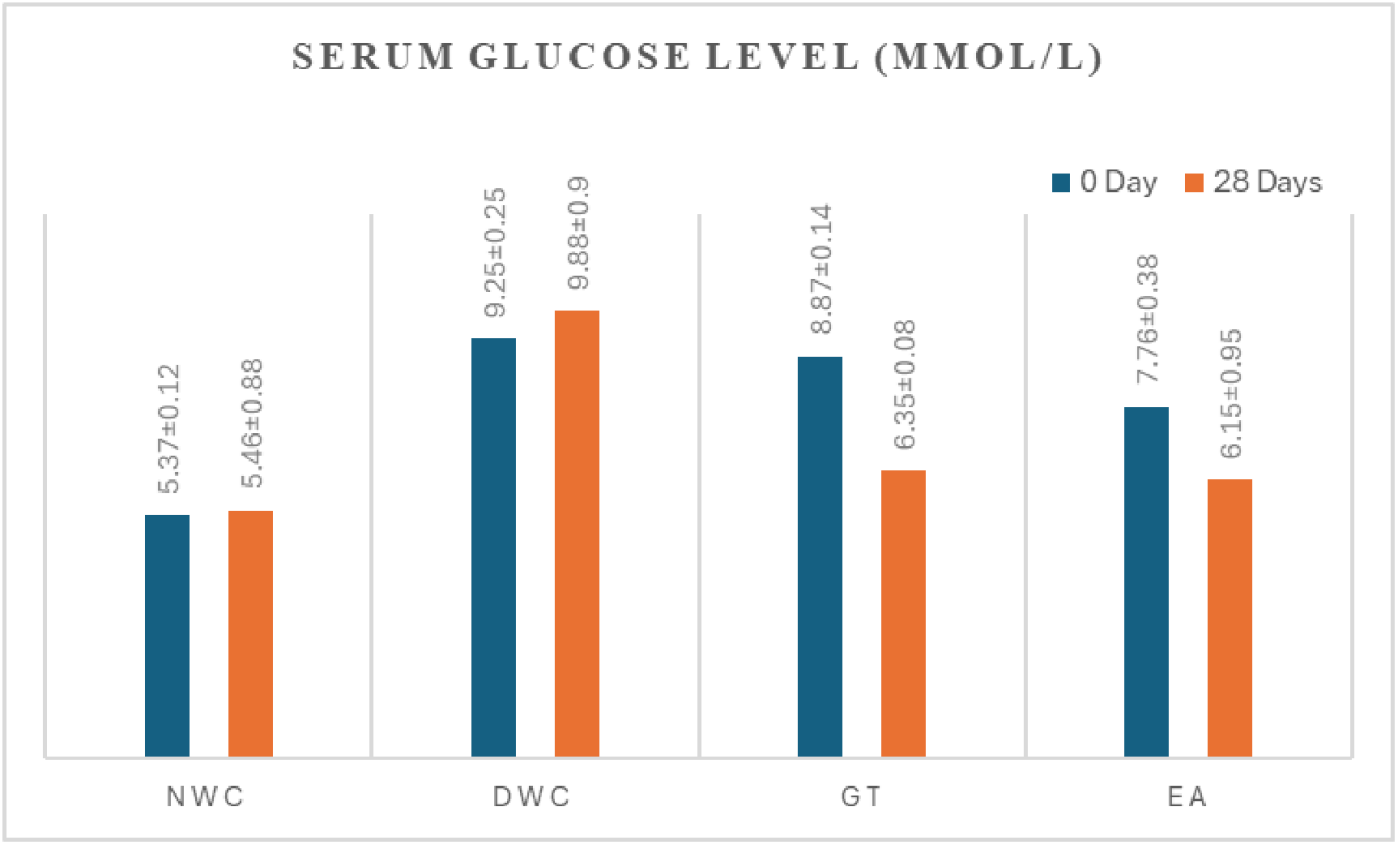
Serum glucose level in different experimental rat groups. Group NWC, DWC, GT, and EA represent normal water control, diabetic water control rat, Gliclazide-treated rat, and *Elaeocarpus angustifolius-treated* rat, respectively. Values are expressed as mean ± standard deviation (M ± SD). Statistical significance among groups was determined using one-way ANOVA, followed by a paired sample t-test for post hoc comparison.

After 28 days of treatment, the serum glucose level of the extract-treated group in rats decreased 21% when compared to the baseline value. The EA-treated group slightly decreased fasting serum glucose (p < 0.013) when compared with the baseline value (0 vs 28 days). Moreover, a non-significant reduction (p > 1.000) of glucose level was found when compared with the GT group. As expected, fasting serum glucose levels of DWC and NWC rats in 28^th^ days increased by 7% and 2%, respectively, compared to the baseline value. Likewise, the fasting serum glucose level of the GT group of rats in 28^th^ days decreased by 28% (Fig 2 and S6 Table). Similarly, previous studies of *Elaeocarpus* species show a significant reduction of serum glucose level in experimental animals [47].

#### 3.7.3 Lipid profile level.

After 28 days of treatment, in a lipid profile, the *Ea-treated* group showed a 5% decrease in triglycerides (TG), 2% decrease in cholesterol, 5% increase in high-density lipoproteins (HDL), and 5% decrease in low-density lipoprotein (LDL), which is quite comparable with standard Gliclazide. Similarly, the *Ea-treated* group shows a significant reduction (p < 0.006) in TG value, a significant reduction (p < 0.034) in cholesterol value and a significant reduction (p < 0.040) in LDL value but HDL shows a significant increase (p > 0.144) respectively when compared to the baseline value (0 vs 28 days). Moreover, a significant reduction (p < 0.001) of TG, HDL, and LDL was found as compared with the GT value (S7 Table). Also, a similar reduction in triglycerides, cholesterol, and low-density lipoproteins and a significant increase in high-density lipoproteins were observed in an earlier report of *Elaeocarpus* species [48].

#### 3.7.4 Glycogen level.

**Fig 3:**
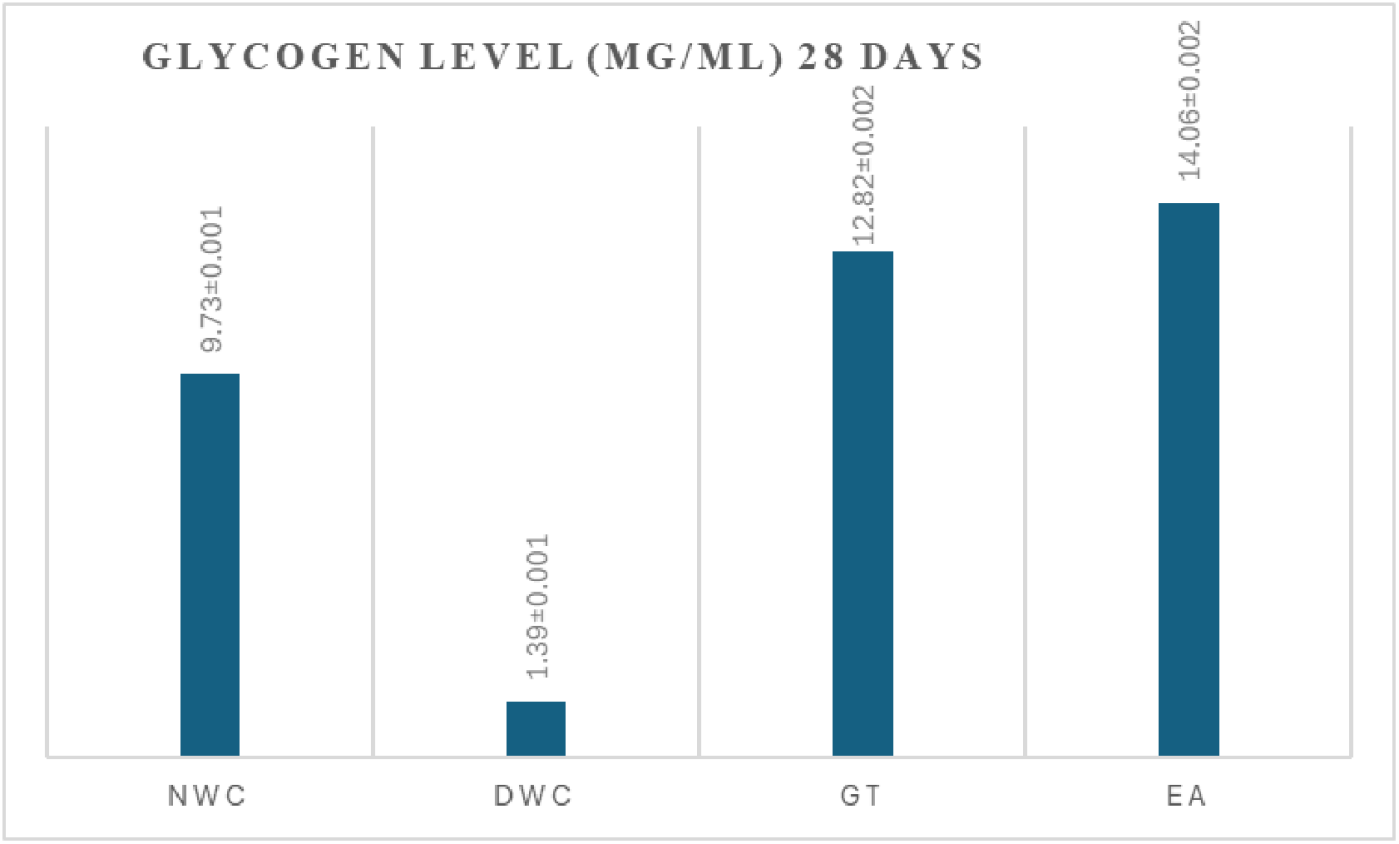
Glycogen content in different experimental rat groups. Group NWC, DWC, GT, and EA represent normal water control, diabetic water control rat, Gliclazide-treated rat, *Elaeocarpus angustifolius-treated* rat, respectively. Values are expressed as mean ± standard deviation (M ± SD). Statistical significance among groups was determined using one-way ANOVA, followed by a paired sample t-test for post hoc comparison.

After 28 Days of treatment, the *Ea* extract-treated group shows a higher glycogen value of 14.06 ± 0.002 mg/mL as compared to DWC treated group with a glycogen value of 1.39 ± 0.002 mg/mL (Fig 3 and S8 Table). Also, the EA-treated group increased significantly (p < 0.001) when compared with the DWC, NWC, and GT treated group.

### 3.8 LC-MS analysis

LC-MS analysis of 100% ethylacetate extract of *E. angustifolius* identified thirteen phytoconstituents, as detailed in Table 1. All compounds were characterized by analysing their spectral information, including molecular mass and retention time, acquired using positive and negative mode electron spray ionization mass spectrometry (ESI-MS) (S4 and S5 Figs), respectively.

**Table 1:** Phytoconstituents identified by liquid chromatography-mass spectrometry on ethylacetate fractions of *E. angustifolius.*

Sl. No	RT (Min)	Identification	Molecular formula	Actual m/z	Experimental m/z [M-H]	Fragments m/z	Error	Library score
1.	0.85	D-saccharic acid	C_6_H_10_O_8_	210.1388	209.0313	85.0304, 71.0149,57.0357	1.1075	78.5
2.	1.38	Gallic acid	C_7_H_6_O_5_	170.1195	169.0239	125.0270, 79.0205,51.0253	1.0956	98.4
3.	4.04	Vanillic acid	C_8_H_8_O_4_	168.1467	167.0330	108.0226, 91.0199, 65.0042	1.1137	65.4
4.	5.21	Methyl gallate	C_8_H_8_O_5_	184.1461	183.0370	124.0258, 78.0156	1.1091	97.1
5.	6.29	Ferulic acid	C_10_H_10_O_4_	194.1840	193.0488	134.0385, 133.0300, 89.0421	1.152	90.6
6.	6.09	Corilagin	C_27_H_22_O_18_	634.4550	633.0807	301.0034, 275.0229	1.3743	100.0
7.	6.90	Ethylgallate	C_9_H_10_O_5_	198.1727	197.0463	124.0178, 78.0119	1.1264	95.5
8.	8.73	Abscisic acid	C_15_H_20_O_4_	264.3210	263.1302	219.1441, 189.0982, 139.0776	1.1908	92.7
9.	14.20	Emodin	C_15_H_10_O_5_	270.2369	269.0468	241.0528, 225.0574, 197.0621	1.1901	96.1
10.	26.99	Ellagic acid	C_14_H_6_O_8_	302.1970	301.0024	284.0006, 245.0130, 229.0123	1.1946	86.7
11.	6.43	Rutin	C_27_H_30_O_16_	610.5175	611.1674	303.0483	0.6499	78.2
12.	7.25	Geraniin	C_41_H_28_O_27_	952.6480	951.0856	933.0735, 301.0018	1.5624	77.3
13.	7.38	Isoquercetin	C_21_H_20_O_12_	464.3763	463.0983	301.0301, 167.0180	1.278	99.0

The mass spectrum of the peak eluted at 0.85 min revealed adduct ions with a *m/z* value of 209.0313 [M-H]^-^. Additionally, a fragment ion was observed at *m/z* 85.0304, which resulted from sequential loss of carboxylic and hydroxyl group to form stabilized acylium ion at *m/z* 85.0304 [OH-CH_2_-CH = CH-C⁺=O]. Through the analysis of mass spectra *m/z* database and relevant scientific literature, D-saccharic acid was determined to be the compound. This compound has been identified in *Elaeocarpus* species [49].

The mass spectrum of the peak eluted at 1.39 min revealed adduct ions with a *m/z* value of 169.0239 [M-H]^-^. Also, a fragment ion was observed at *m/z* 125.0270, which resulted from sequential loss of CO_2_ (44 Da), represented as [M-H-CO_2_]. Such findings and analysis of databases confirm the compound as Gallic acid. Similar findings were aligned with a previous report on various species of *Elaeocarpus* [50].

The analyte’s mass spectrum eluted at 3.04 min, detected adduct ions with a *m/z* value of 167.0330 [M-H]^-^. Moreover, a fragment ion with a *m/z* value of 108.0226 was found, which resulted from decarboxylation and demethylation of vanillic acid. Through the analysis, scientific literature, and earlier reports on *Elaeocarpus,* the compound was determined to be vanillic acid [50].

The mass spectral peak appeared at 5.23 min of retention time, revealing adduct ions with a *m/z* value of 183.0370 [M-H]^-^. Furthermore, a fragment ion was observed at *m/z* 124.0258, which resulted from decarboxylation and demethylation, represented as [M-H-CO_2_-CH3]. These spectral characteristics, supported by scientific literature and database analysis, identified the compound as Methyl gallate. Similar findings were also reported in earlier findings on *Elaeocarpus* species [51].

The peak appeared at 6.29 min showed a mass spectrum of an ion with a *m/z* value of 193.05 [M-H]^-^. Additionally, a fragment ion was observed at *m/z* 134.04, likely due to decarboxylation and demethylation, labelled as [M-H-CO_2_-CH_3_]. Several databases and scientific literature supported this to be the compound Ferulic acid. Such findings were aligned with previous reports on *Elaeocarpus* species [52].

The mass spectrum of the peak eluted at 6.09 min revealed adduct ions with a *m/z* value of 633.0807 [M-H]^-^. Similarly, a fragment of ion was reported at the *m/z* value of 301.0034, likely due to the formation of a dimer of gallic acid (C_14_H_6_O_8_). By comparing these findings with previous reports on *Elaeocarpus* species, the compound was matched with Corilagin. Based on such similarity, this compound was reported as Corilagin [53].

Analysis of the peak with retention time of 6.90 min revealed the adduct ions with a *m/z* value of 197.0463 [M-H]^-^. In addition, a fragment ion at m/z value of 124.0178 was also observed, which is due to loss of ethyl group (CH_2_CH_3_) and decarboxylation (44 Da), labelled as [M-H- CH_2_CH_3_-CO_2_]. Similar findings and through a literature survey on *Elaeocarpus* species, this compound was confirmed to be Ethyl gallate [54].

The mass spectrum of the peak eluted at 8.73 min displays adduct ions with a *m/z* value of 263.1302 [M-H]^-^. Likewise, a fragment of ion was reported at the *m/z* value of 219.1441, due to decarboxylation represented as [M-H- CO_2_]. Such findings revealed the compound to be abscisic acid, which was confirmed by several literature surveys and available databases on *Elaeocarpus* species [49].

The mass spectral peak appeared at 14.20 min of retention time, revealing the *m/z* value of 269.0468 [M-H]^-^. Also, fragments of ions were shown at *m/z* value of 241.0528 due to loss of (CO-28 Da) and at m/z value of 225.06, likely due to demethylation (CH_3_–15 Da). By comparing phytoconstituents reported on various species of *Elaeocarpus,* the compound was confirmed as emodin [49].

The peak appeared at 26.99 min of retention time, revealing the mass spectrum at *m/z* value of 301.0024 [M-H]^-^. In addition, a fragment of ion was observed at the *m/z* value of 284.0006, likely due to loss of water (H_2_O). Through the analysis of databases and related scientific literature, the compound was confirmed as ellagic acid, which is also reported in earlier reports on *Elaeocarpus* species [55].

The mass spectrum of the peak eluting at 6.43 min exhibits adduct ions at the *m/z* value of 611.1674 [M-H]^-^. Additionally, a fragment ion at *m/z* of 303.0483 was detected, attributed to the sequential loss of sugar moiety (C_12_H_20_O_9_ −308 Da). These results, together with database comparison, confirmed the compound as rutin, aligned with previously reported data on *Elaeocarpus* species [54].

The peak observed at 6.26 min of retention time showed a mass spectrum at *m/z* value of 951.0856 [M-H]^-^ and a fragment ion at *m/z* 933.0735, corresponding with the loss of water (H_2_O). Database comparison confirmed the compound as geraniin, which is also reported in prior reports of various species of *Elaeocarpus* [56]*.*

The mass spectrum eluted at 7.38 min showed adduct ions at the *m/z* value of 463.0983 [M-H]^-^. Similarly, a fragment of ion was observed at *m/z* 301.0301, likely due to the formation of quercetin derivatives. Through database analysis and literature, the compound was determined to be isoquercetin. This compound has been identified in various species of *Elaeocarpus* [57].

### 3.9 Computational analysis

#### 3.9.1 Molecular docking analysis.

After the LC-MS study, molecular docking was carried out among the identified compounds and the target proteins. The active site residues for alpha-glucosidase with glucose molecules were **HIS122, ARG213, ASP215, ASP69, GLU277, HIS351, ARG442, ASP352**, and for alpha amylase with acarbose molecules active site residues were T**YR62, GLN63, THR163, THR163, GLY164, ASN105, ALA106, VAL107, TRP59, HIS101, GLU233, ARG195, ASP300, and HIS29**9. Corilagin had the lowest binding affinity of −10.3 kcal/mol, lower than the reference ligand −8.4 kcal/mol for alpha amylase (Table 2). Whereas Rutin had the lowest binding affinity of −10.0 kcal/mol, surpassing the reference ligand −9.1 kcal/mol for alpha-glucosidase. Both show lower binding affinity for the target proteins, which means greater stability at the orthosteric sites.

**Table 2:** Molecular docking score of different constituents against α-amylase and α-glucosidase.

S. N	PubChem ID	Compound Name	Binding affinity with α-amylase (Kcal/mol)	Binding affinity with α-glucosidase (Kcal/mol)
1	73568	D-Saccharic acid	−6.3	−6.0
2	5280805	Gallic acid	−6.3	−6.4
3	5280804	Vanillic acid	−5.6	−6.1
4	3220	Methyl gallate	−6.4	−6.5
5	5281855	Ferulic aci d	−6.6	−6.6
6	3001497	Corilagin	−10.3	−9.6
7	5280896	Ethyl gallate	−6.4	−6.5
8	445858	Abscisic acid	−7.0	−6.8
9	7428	Emodin	−8.4	−8.3
10	13250	Ellagic acid	−8.7	−8.1
11	370	Rutin	−9.6	−10.0
12	8468	Geraniin	−9.6	−8.1
13	78997	Isoquercetin	−8.6	−9.4
14	79025	Alpha-D-Glucose (Native ligand)	–	−5.9
15	41774	Glucobay (Native ligand)	−10.4	–
16	445421	Alpha-Acarbose (Reference drug)	−8.4	−9.1

**Fig 4:**
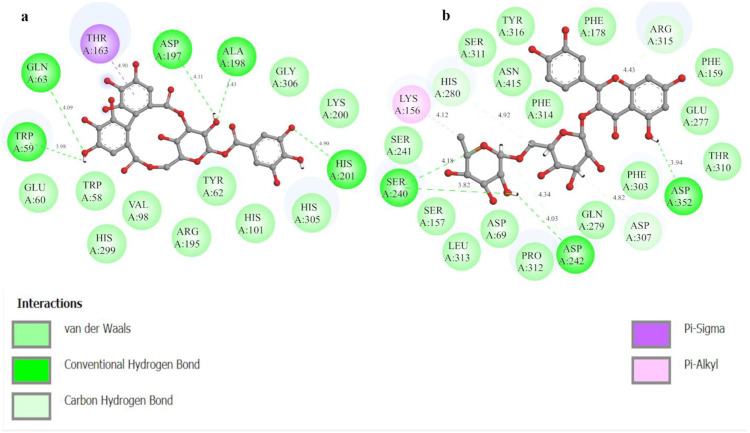
A two-dimensional representation of protein-ligand interaction. **a)** Corilagin-alpha amylase **b)** Rutin-alpha glucosidase.

The alpha amylase-Corilagin complex interacts with key residues of amino acids such as ASP197 (4.11 Å), ALA198 (3.43 Å), HIS201 (4.90 Å), GLN63 (4.09 Å), and TRP59 (3.98 Å) through hydrogen bonding (Fig 4a). Additional interactions include Van der Waals’ force of attraction (GLY306, LYS200, HIS101, GLU60, HIS305, ARG195, HIS299, VAL98, TRP58, TYR62), and Pi-Sigma (THR163). Similarly, the alpha-glucosidase-Rutin complex forms hydrogen bonds with SER240 (4.18 Å and 3.82 Å), ASP242 (4.03 Å), and ASP352 (3.94 Å) amino acid residues (Fig 4b). Other interactions include Pi-Alkyl (LYS156), Carbon Hydrogen Bond (ARG315, ASP307, ASP242, HIS280), and Van der Waals’ (SER241, SER311, ASN415, PHE314, TYR316, PHE178, PHE159, GLU277, THR310, PHE303, GLN279, PRO312, ASP69, LEU313, SER157). Previous investigations through an identical receptor reported comparable active site residues, suggesting that the calculations were consistent [58,59].

#### 3.9.2 Analysis of molecular dynamics.

**Fig 5:**
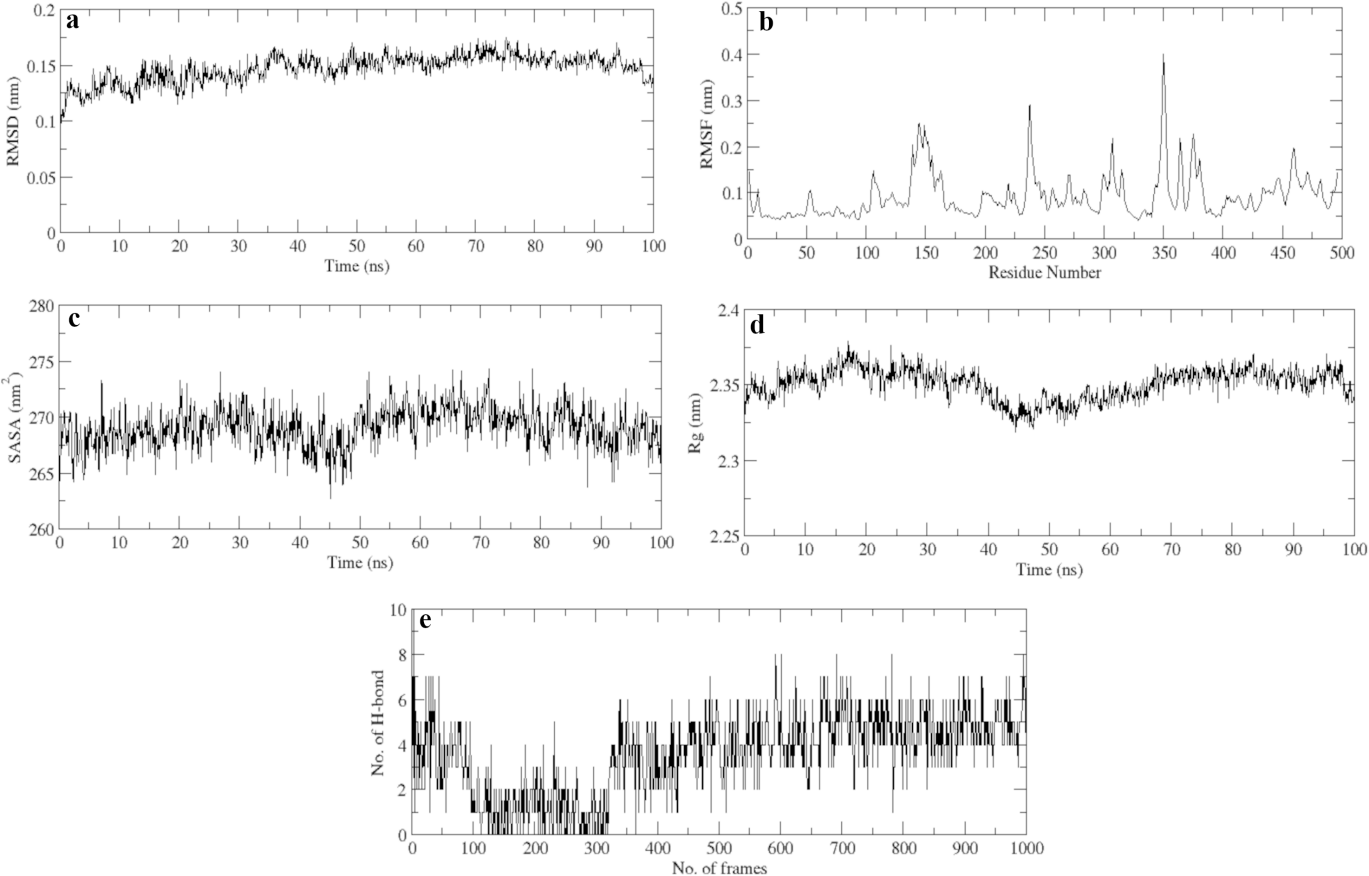
The plot obtained from 100 ns MDSa) RMSD with respect to protein backbone b) RMSF with respect to per residue c) SASA of protein d) Rg of protein e) H-bond count.

Corilagin exhibits the highest binding affinity to α-amylase among the docked isolated compounds with both proteins. It was further investigated through a 100 ns molecular dynamics simulation to ensure that it has the highest binding affinity to alpha amylase among the docked isolated compounds with both proteins. Additionally, a 100 ns molecular dynamics simulation was conducted to ensure equilibrium of relevant system properties. The protein-backbone RMSD remained smooth at 1.469 ± 0.133 Å, indicating consistency over time (Fig 5a). Most residues had RMSF values less than 0.92 ± 0.476 Å, indicating very little fluctuation during simulation (Fig 5b). The lower RMSF value indicates that the complex is less flexible, reflecting its greater stability [60]. The protein’s SASA was used to determine the entire wettable area of the protein throughout MDS, and the complex’s SASA value was found to be 269.085 ± 1.872 nm^2^ (Fig 5c). The trajectory of complex formation for SASA showed less fluctuation, indicating that the complex is stable with a uniform surface area of the protein interaction with the solvent [61]. The radius of gyration (Rg) measures the compactness of the protein-ligand complex, with lower Rg values indicating a more compact structure [62]. The Rg calculation revealed a stable and smooth trajectory at 23.5 ± 0.106 Å, indicating the stability of the complex (Fig 5d). The total amount of hydrogen bonds was thoroughly monitored to over 1000 frames of MDS trajectories (Fig 5e). The continued stability of six hydrogen bonds after 350 frames, with the highest ten hydrogen bonds, indicates that the ligands are stable in their docked pose, with no significant RMSD shifts observed over the MDS period [63,64]. All these parameters show that the complex is geometrically stable throughout the MDS.

#### 3.9.3 Binding free energy estimations.

**Fig 6:**
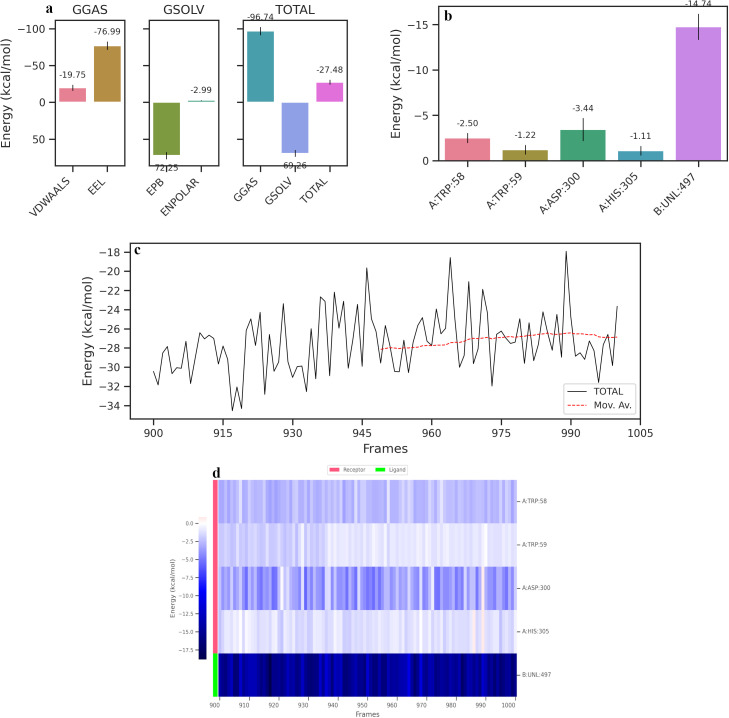
The plot showinga) Free energy contributions of various energies to the binding energy b) The active amino acids and ligands contribute to binding free energy c) binding free energy change for complex d) A heat map displaying residue-specific contributions per frame.

The change in binding free energy measures both the feasibility and spontaneity of complex formation. A lower binding free energy change (ΔG_BFE_) indicates increased complex stability. The binding energy was found to be −27.48 ± 3.15 kcal/mol (Table 3). Complex formation was characterized by negative ΔG_BFE_ values, indicating a spontaneous reaction (Fig 6c). The key amino acid residues that contributed to the free energy were ASP300, TRP58, TRP59, HIS305, and the ligand (Fig 6b). These outcomes were backed up by a heat map of the complex that visually represented residue contributions (Fig 6d). Darker blue colours on the heatmap represent residues that contribute more energy, and lighter, faded colours indicate minimal involvement, verifying these residues’ critical roles in ligand binding [61].

**Table 3:** The components of the complex’s binding free energy (kcal/mol) vary.

ΔE_VDW_	ΔE_EL_	ΔE_PB_	ΔE_NPOLAR_	ΔG_GAS_	ΔG_SOLV_	ΔG_BFE_
−19.75 ± 4.07	−76.99 ± 5.7	72.25 ± 4.81	−2.99 ± 0.31	−96.74 ± 5.67	69.26 ± 4.68	−27.48 ± 3.15

#### 3.9.4 ADMET analysis.

The top-performing candidate, Corilagin (class 5), has an LD_50_ of 2260 mg/kg. The hit candidate and acarbose both were immunotoxic; however, acarbose also exhibits hepatotoxicity, and other ADMET properties were discussed, in which neither follows Lipinski’s rule of five (3 violations) that was due to the high molecular mass and more hydrogen bond donors (Table 4) [65]. The human intestinal absorption rate for Corilagin was found to be 58.501%, which was higher than that of acarbose, 4.172%. The logBB < −1 and logPS < −3 values indicate failing to get inside the central nervous system and poor brain distribution [66]. Cytochrome enzymes, which are crucial to xenobiotic metabolism, were not inhibited by the hit compound [67]. Overall, hit candidates exhibited acceptable ADMET properties.

**Table 4:** Drug-likeness properties of Corilagin and the reference Drug.

Properties	Corilagin	Acarbose
Water solubility	−2.892	−1.482
Caco2 permeability	−1.337	−0.481
Intestinal absorption (human)	58.509	4.172
Skin permeability	−2.735	−2.735
BBB permeability	−2.828	−1.717
CNS permeability	−5.044	−6.438
CYP2D6 substrate	No	No
CYP3A4 substrate	No	No
CYP1A2 inhibitor	No	No
CYP2C19 inhibitor	No	No
CYP2C9 inhibitor	No	No
CYP2D6 inhibitor	No	No
CYP3A4 inhibitor	No	No
Total Clearance	0.229	0.428
Renal OCT2 substrate	Yes	No
Hepatotoxicity	No	Yes
Immunotoxicity	Yes	Yes
Carcinogenicity	No	No
Cytotoxicity	No	No
Mutagenicity	No	No
Lipinski rule	3 violations	3 violations

## 4. Conclusion

The findings revealed that the methanol extract of *E. angustifolius* bark contained higher levels of total flavonoid content and total phenolic content, attributed to the diverse bioactive secondary metabolites present. It was observed that crude and fraction solutions exhibit superior antioxidant and antidiabetic activity against α-glucosidase and α-amylase, which demonstrates therapeutic potential for the treatment and management of diabetes mellitus and its related complications. Additionally, in vivo antidiabetic activity suggests that *E. angustifolius* bark extract effectively reduces glycemic status and improves dyslipidemia by lowering the lipid profile (TG, cholesterol, LDL) and increasing HDL cholesterol levels. Computational evaluation of the thirteen compounds identified using LC-MS was carried out with alpha-glucosidase and alpha-amylase enzymes, considering both active and allosteric pockets. It support to the *in vitro* and *in vivo* findings and further suggests that Corilagin formed a stable complex with an alpha amylase at its active site, indicating it as a potential candidate for further validation. These findings suggest that extracts from *E. angustifolius* bark could be valuable resources for the exploration of new, potent bioactive compounds, which can be isolated as pure compounds.

## Supporting information

S1 Fig The binding interaction between the initially docked co-crystallized ligand (yellow, obtained from the PDB) and the re-docked ligand (green) results in an RMSD below 2A^°^. (a) α-glucosidase, (b) α-amylase.
(TIF)

S2 Fig Calibration curve of standard gallic acid (TPC). (TIF)

S3 Fig Calibration curve of standard quercetin (TFC). (TIF)

S4 Fig LC-MS in positive ion- mode for ethylacetate fraction of *E. angustifolius.* (TIF)

S5 Fig LC-MS in negative ion- mode for ethylacetate fraction of *E.angustifolius.* (TIF)

S1 Table Qualitative phytochemical analysis of the methanol extract of *E. angustifolius.* (DOCX)

S2 Table *In vitro* antioxidant activity of extract and different fractions of *E. angustifolius.* (DOCX)

S3 Table *In vitro* α-glucosidase enzyme inhibition assay of *E. angustifolius.* (DOCX)

S4 Table *In vitro* α-amylase enzyme inhibition assay of *E. angustifolius* bark extract and fractions. (DOCX)

S5 Table Effect of *E. angustifolius* bark methanol extract on the body weight of STZ-induced diabetic rats. (DOCX)

S6 Table Effect of *E. angustifolius* methanol extract on serum glucose level. (DOCX)

S7 Table Effect of methanol extract of *E. angustifolius* on lipid profile. (DOCX)

S8 Table Effect of *E. angustifolius* methanol extract on hepatic glycogen content. (DOCX)
