# Supplementary figures and images for: Biochemical and antidiabetic properties of Elaeocarpus angustifolius Blume: In vitro, In vivo, and In silico insights
Source: PLoS One. 2026 Jun 8;21(6):e0349796. doi: 10.1371/journal.pone.0349796 (PMC13245756; doi:10.1371/journal.pone.0349796)

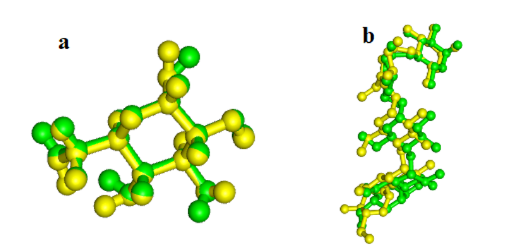

Supplement: S1 Fig — (a) α-glucosidase, (b) α-amylase. (TIF) [file pone.0349796.s001.tif]

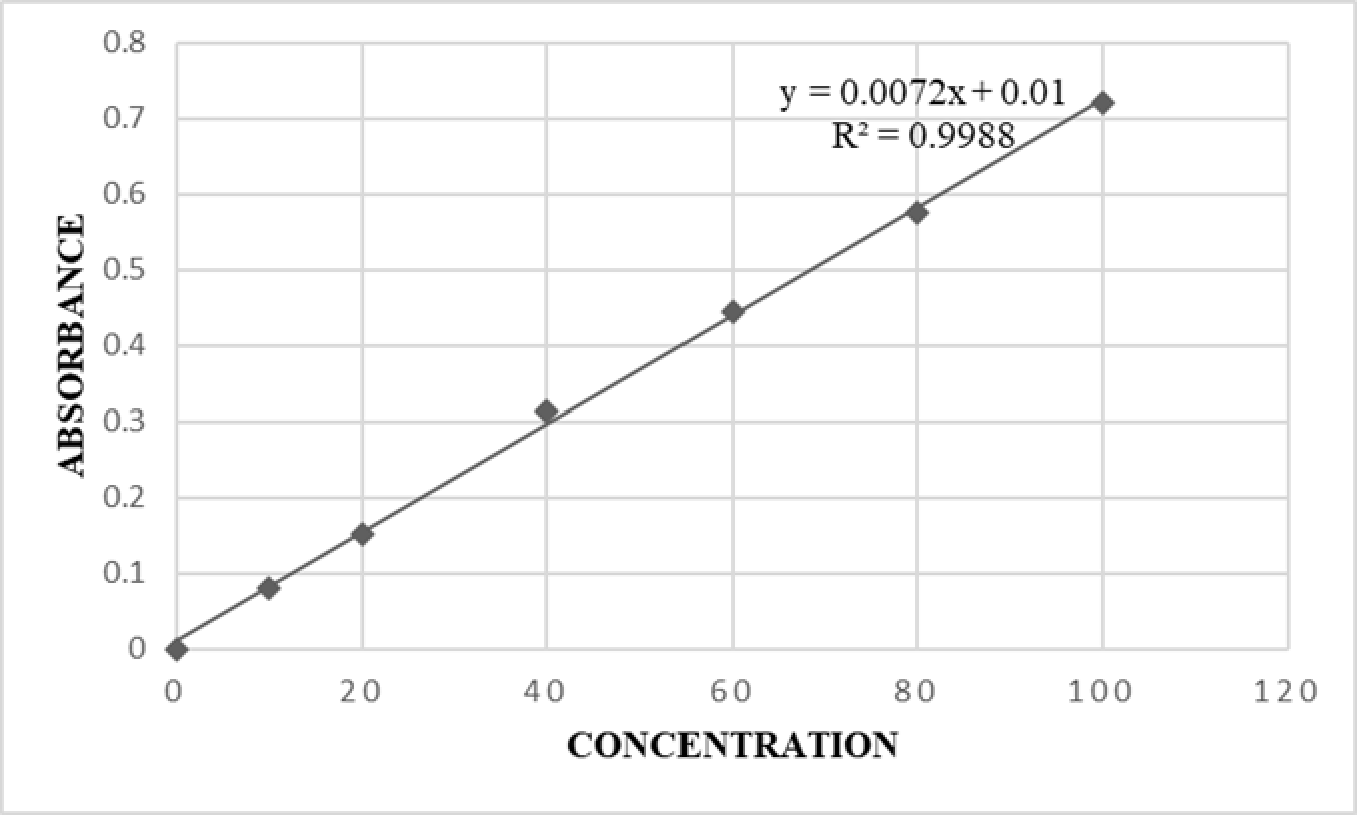

Supplement: S2 Fig — (TIF) [file pone.0349796.s002.tif]

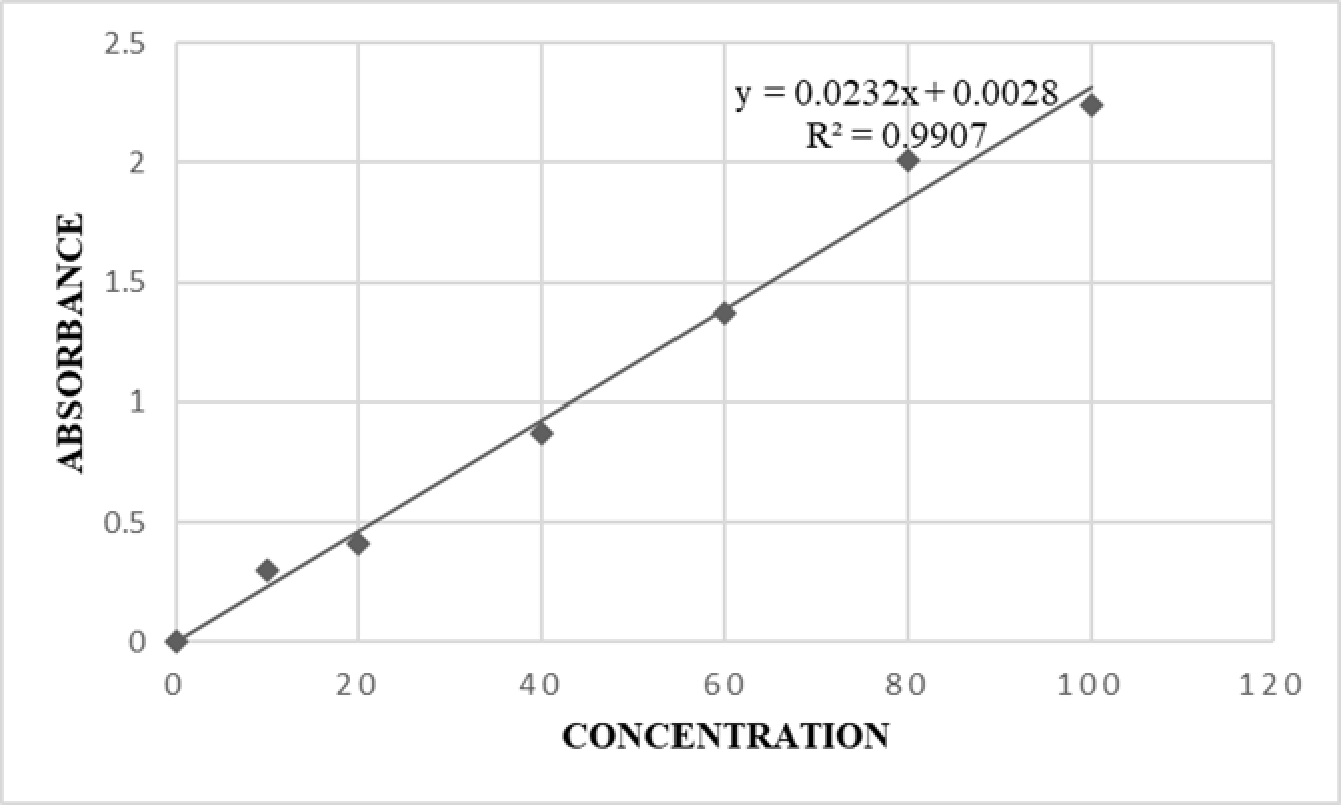

Supplement: S3 Fig — (TIF) [file pone.0349796.s003.tif]

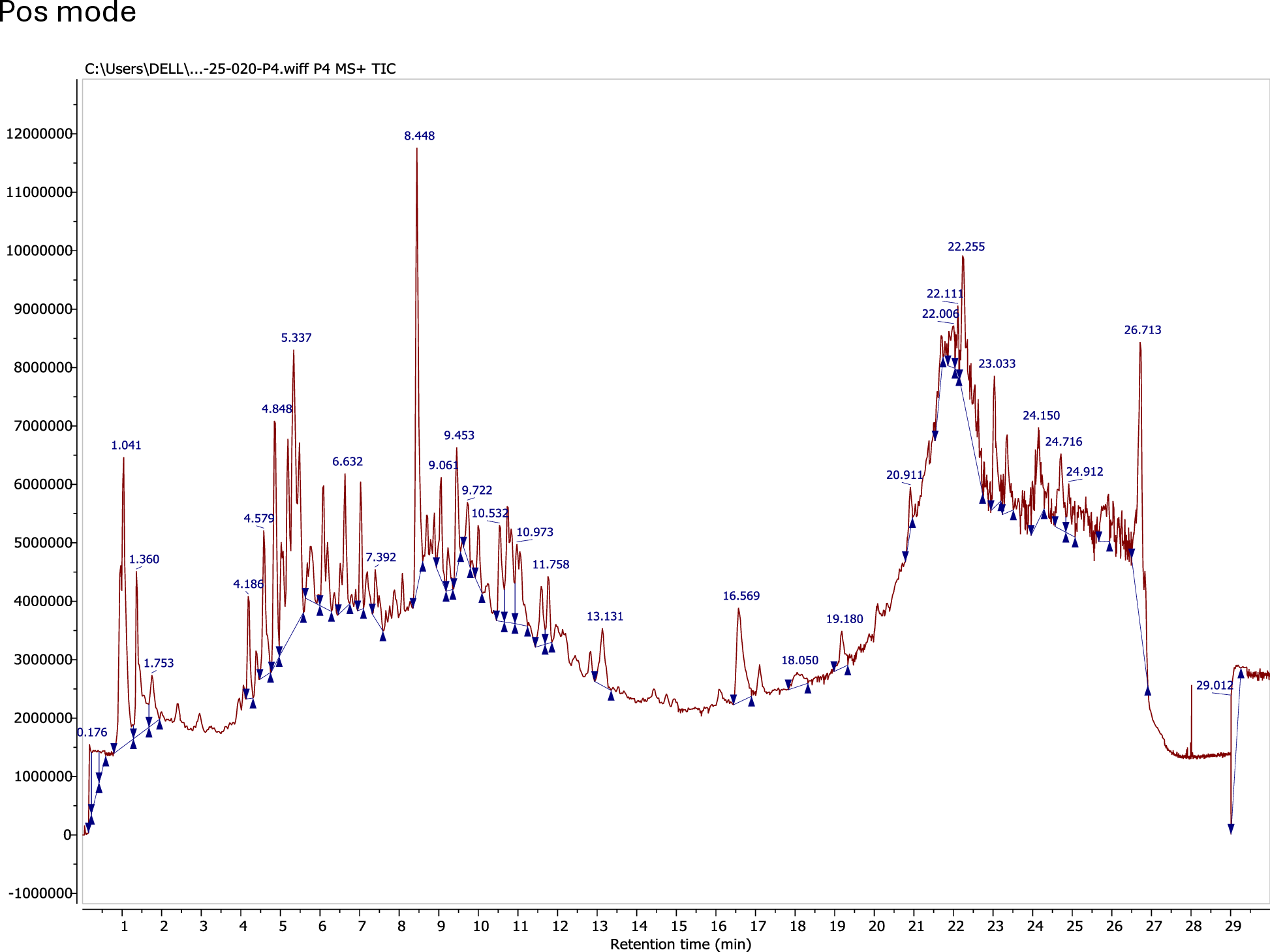

Supplement: S4 Fig — (TIF) [file pone.0349796.s004.tif]

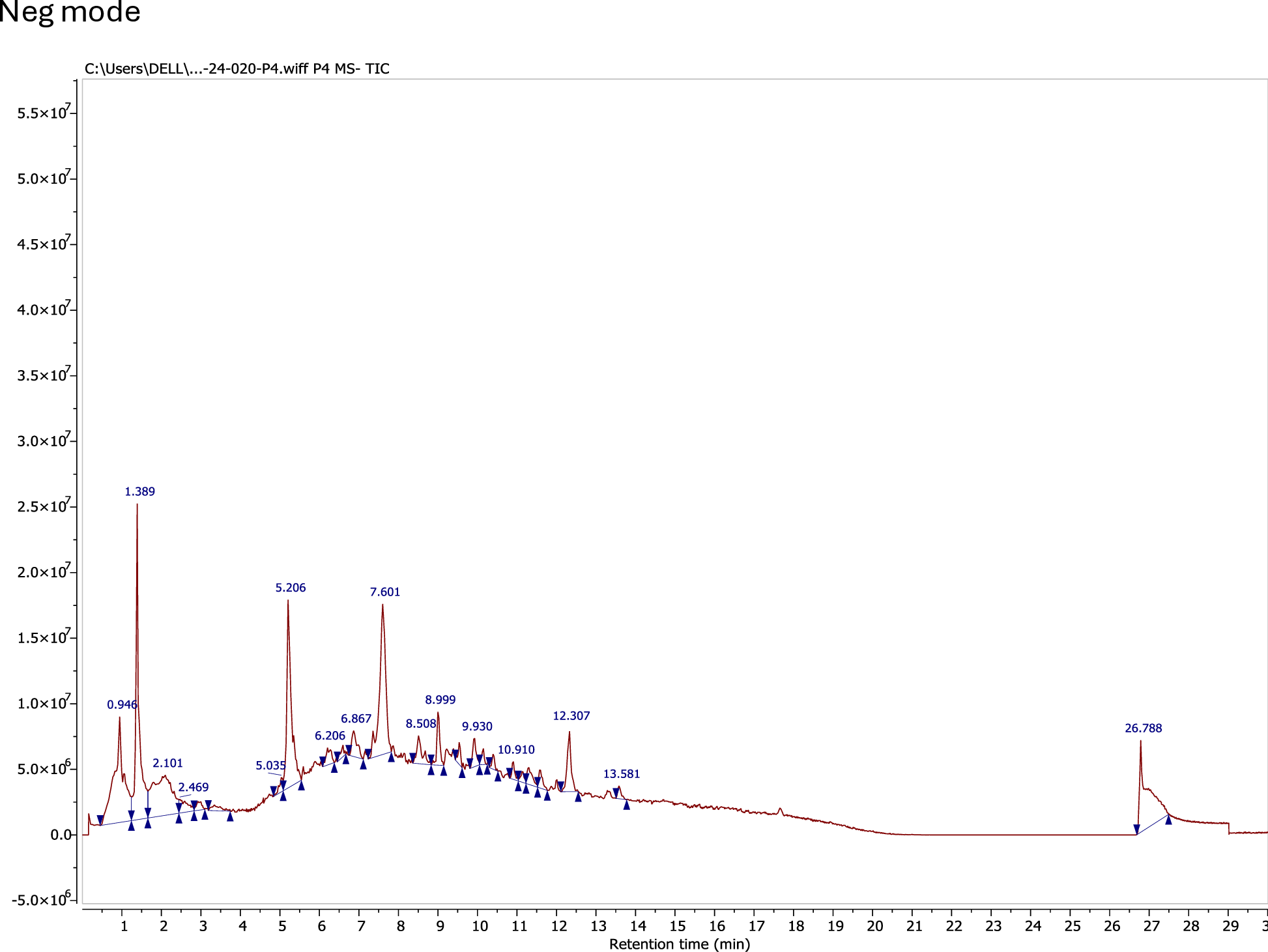

Supplement: S5 Fig — (TIF) [file pone.0349796.s005.tif]
