# Supplementary material for: Biochemical and antidiabetic properties of Elaeocarpus angustifolius Blume: In vitro, In vivo, and In silico insights
Source: PLoS One. 2026 Jun 8;21(6):e0349796. doi: 10.1371/journal.pone.0349796 (PMC13245756; doi:10.1371/journal.pone.0349796)
Supplement: S1 Table — (DOCX) [file pone.0349796.s006.docx]

S1 Table: Qualitative phytochemical analysis of methanol extract of *E.angustifolius.*

| **S. N** | **Phytochemicals** | **Barks** |
| --- | --- | --- |
| **1.** | Alkaloids | **+** |
| **2.** | Flavonoids | **+** |
| **3.** | Terpenoids | **+** |
| **4.** | Reducing sugar | **+** |
| **5.** | Phenols | **+++** |
| **6.** | Saponins | **+** |
| **7.** | Carbohydrates | **+** |
| **8.** | Volatile oil | **+** |
| **9.** | Glycosides | **+** |
