# Supplementary material for: Biochemical and antidiabetic properties of Elaeocarpus angustifolius Blume: In vitro, In vivo, and In silico insights
Source: PLoS One. 2026 Jun 8;21(6):e0349796. doi: 10.1371/journal.pone.0349796 (PMC13245756; doi:10.1371/journal.pone.0349796)
Supplement: S2 Table — (DOCX) [file pone.0349796.s007.docx]

S2 Table: *In vitro* antioxidant activity of extract and different fractions of *E.angustifolius.*

| **S.N** | **Sample** | **IC_50_ (µg/mL) Bark** |
| --- | --- | --- |
| 1. | Methanol extract | 1.48 ± 0.39 |
| 2. | Hexane fraction | 6.48 ± 1.11 |
| 3. | DCM fraction | 13.46 ± 0.00 |
| 4. | Ethylacetate fraction | 1.47 ± 0.37 |
| 5. | Aqueous fraction | 1.48 ± 0.28 |
| 6. | Quercetin | 4.87 ± 0.16 |

Values are the mean of three experiments ± standard error mean (SEM)
