# Supplementary material for: Biochemical and antidiabetic properties of Elaeocarpus angustifolius Blume: In vitro, In vivo, and In silico insights
Source: PLoS One. 2026 Jun 8;21(6):e0349796. doi: 10.1371/journal.pone.0349796 (PMC13245756; doi:10.1371/journal.pone.0349796)
Supplement: S3 Table — (DOCX) [file pone.0349796.s008.docx]

S3 Table: *In vitro* α-glucosidase enzyme inhibition assay of *E. angustifolius.*

| **S.N** | **Sample (bark)** | **IC_50_ (****µg/mL)** |
| --- | --- | --- |
| 1. | Methanol extract | 0.79 ± 0.13 |
| 2. | Hexane fraction | 1.73 ± 0.02 |
| 3. | DCM fraction | 16.97 ± 0.22 |
| 4. | Ethylacetate fraction | 1.79 ± 0.29 |
| 5. | Aqueous fraction | 1.46 ± 0.30 |
| 6. | Acarbose | 13.51 ± 0.22 |

Values are the mean of three experiments ± standard error mean (SEM)
