# Supplementary material for: Biochemical and antidiabetic properties of Elaeocarpus angustifolius Blume: In vitro, In vivo, and In silico insights
Source: PLoS One. 2026 Jun 8;21(6):e0349796. doi: 10.1371/journal.pone.0349796 (PMC13245756; doi:10.1371/journal.pone.0349796)
Supplement: S4 Table — (DOCX) [file pone.0349796.s009.docx]

S4 Table:  *In vitro* α-amylase enzyme inhibition assay of *E. angustifolius* bark extract and fractions.

| **S. N** | **Sample (bark)** | **IC_50_ (µg/mL)** |
| --- | --- | --- |
| **1.** | Methanol extract | 3.48 ± 0.00 |
| **2.** | Hexane extract | 10.33 ±0.15 |
| **3.** | DCM extract | 23.53 ±0.18 |
| **4.** | Ethylacetate extract | 9.36 ± 0.38 |
| **5.** | Aqueous extract | 4.79 ±0.06 |
| **6.** | Acarbose | 25.30 ± 0.85 |

Values are the mean of three experiments ± standard error mean (SEM)
