# Supplementary material for: Biochemical and antidiabetic properties of Elaeocarpus angustifolius Blume: In vitro, In vivo, and In silico insights
Source: PLoS One. 2026 Jun 8;21(6):e0349796. doi: 10.1371/journal.pone.0349796 (PMC13245756; doi:10.1371/journal.pone.0349796)
Supplement: S5 Table — (DOCX) [file pone.0349796.s010.docx]

S5 Table: Effect of *E. angustifolius* bark methanol extract on body weight of STZ-induced diabetic rats.

| **Group** | **Body weight (g)** | | | | |
| --- | --- | --- | --- | --- | --- |
|  | 0 day | 7 days | 14 days | 21 days | 28 days |
| NWC(n=5) | 242±1.92 (100%) | 244±0.84 | 255±1.30 | 262±1.41 | 266±0.84 (110%) |
| DWC(n=5) | 233±2.45 (100%) | 235±3.11 | 242±2.30 | 242±1.22 | 242±1.14 (104%) |
| GT(n=5) | 244± 2.74(100%) | 248±1.14 | 246±2.17 | 243±2.17 | 239±3.21 (98%) |
| *Elaeocarpus angustifolius* (n=6) | 156±3.27 (100%) | 161±3.43 | 164±2.32 | 183±1.47 | 191±3.37 (122%) |
| **One way ANOVA** | | | | | |
| NWC VS DWC | 0.001 | 0.001 | 0.001 | 0.001 | 0.001 |
| NWC VS GT | 1.000 | 0.027 | 0.001 | 0.001 | 0.001 |
| NWC VS *Elaeocarpus angustifolius* | 0.001 | 0.001 | 0.001 | 0.001 | 0.001 |
| DWC VS GT | 0.001 | 0.001 | 0.032 | 1.000 | 0.448 |
| DWC VS *Elaeocarpus angustifolius* | 0.001 | 0.001 | 0.001 | 0.001 | 0.001 |
| GT VS *Elaeocarpus angustifolius* | 0.001 | 0.001 | 0.001 | 0.001 | 0.001 |
| **Paired sample t-test** | | | | | |
| Group | 0 day vs. 28 days | | | | |
| NWC | 0.001 | | | | |
| DWC | 0.003 | | | | |
| GT | 0.05 | | | | |
| *Elaeocarpus angustifolius* | 0.001 | | | | |

Group NWC, DWC, GT, and EA represent normal water control, diabetic water control rat, Gliclazide-treated , and *Elaeocarpus angustifolius-treated* rat, respectively. Data presented as mean ± standard deviation (M ± SD). Statistical comparison between groups was performed using one-way ANOVA.
