# Supplementary material for: Biochemical and antidiabetic properties of Elaeocarpus angustifolius Blume: In vitro, In vivo, and In silico insights
Source: PLoS One. 2026 Jun 8;21(6):e0349796. doi: 10.1371/journal.pone.0349796 (PMC13245756; doi:10.1371/journal.pone.0349796)
Supplement: S6 Table — (DOCX) [file pone.0349796.s011.docx]

S6 Table: Effect of *E. angustifolius* methanol extract on serum glucose level.

| **Group** | **Fasting serum glucose level (mmol/L)** | |
| --- | --- | --- |
|  | 0 day | 28^th^ days |
| NWC | 5.37 ± 0.12 (100%) | 5.46 ± 0.88 (102%) |
| DWC | 9.25 ± 0.25 (100%) | 9.88 ± 0.90 (107%) |
| GT | 8.87 ± 0.14 (100%) | 6.35 ± 0.08 (72%) |
| *Elaeocarpus angustifolius* | 7.76 ± 0.38 (100%) | 6.15 ± 0.95 (79%) |
| **One-way ANOVA** | | |
| NWC VS DWC | 0.001 | 0.001 |
| NWC VS GT | 0.001 | 0.093 |
| NWC VS *Elaeocarpus angustifolius* | 0.001 | 0.259 |
| DWC VS GT | 0.182 | 0.001 |
| DWC VS *Elaeocarpus angustifolius* | 0.001 | 0.001 |
| GT VS *Elaeocarpus angustifolius* | 0.001 | 1.000 |
| **Paired sample t-test** | | |
| Group | 0 day vs. 28^th^ days | |
| NWC | 0.302 | |
| DWC | 0.004 | |
| GT | 0.001 | |
| *Elaeocarpus angustifolius* | 0.013 | |

Group NWC, DWC, GT, and EA, represent normal water control, diabetic water control rat, Gliclazide treated, *Elaeocarpus angustifolius* treated rat respectively. Data presented as mean±standard deviation (M±SD). Statistical comparison between groups was performed using one-way ANOVA and paired sample t-test.
