# Supplementary material for: Biochemical and antidiabetic properties of Elaeocarpus angustifolius Blume: In vitro, In vivo, and In silico insights
Source: PLoS One. 2026 Jun 8;21(6):e0349796. doi: 10.1371/journal.pone.0349796 (PMC13245756; doi:10.1371/journal.pone.0349796)
Supplement: S7 Table — (DOCX) [file pone.0349796.s012.docx]

S7 Table: Effect of methanol extract of *E. angustifolius* on lipid profile.

| **GROUP** | **TG (mg/dl)** | | **Cholesterol (mg/dl)** | | **HDL (mg/dl)** | | **LDL (mg/dl)** | |
| --- | --- | --- | --- | --- | --- | --- | --- | --- |
|  | 0 day | 28^th^ days | 0 day | 28^th^ days | 0 day | 28^th^ days | 0 day | 28^th^ days |
| NWC | 106.24±1.90  (100%) | 105.40±1.52  (99%) | 53.50± 2.28  (100%) | 53.80±1.48  (100%) | 25.34±1.23 (100%) | 27.13±0.84  (107%) | 17.17±0.30 (100%) | 17.18±0.19  (100%) |
| DWC | 128.80±3.99 (100%) | 148.67±4.25  (115%) | 75.35± 1.38 (100%) | 77.50±1.33  (103%) | 28.80±1.30  (100%) | 26.40±1.68  (91%) | 22.51± 1.19 (100%) | 24.53±0.91  (109%) |
| GT | 145.42±2.92  (100%) | 126.0±0.02  (86%) | 73.85±1.85  (100%) | 65.49±1.88  (89%) | 26.24±1.00  (100%) | 29.00±1.08  (111%) | 19.56±1.38  (100%) | 11.86±0.65  (61%) |
| *Elaeocarpus angustifolius* | 87.29±2.20 (100%) | 83.37±3.82  (95%) | 93.50±1.549 (100%) | 91.32±1.0  (98%) | 35.88±0.71 (100%) | 36.48±0.27  (102%) | 40.16±1.67  (100%) | 38.16±1.16  (95%) |
| **One way ANOVA** | | | | | | | | |
| NWC VS DWC | 0.001 | 0.001 | 0.001 | 0.001 | 0.001 | 1.000 | 0.001 | 0.001 |
| NWC VS GT | 0.001 | 0.001 | 0.001 | 0.001 | 1.000 | 0.076 | 0.052 | 0.001 |
| NWC VS *Elaeocarpus angustifolius* | 0.001 | 0.001 | 0.001 | 0.001 | 0.001 | 0.001 | 0.001 | 0.001 |
| DWC VS GT | 0.001 | 0.001 | 1.000 | 0.001 | 0.009 | 0.007 | 0.011 | 0.001 |
| DWC VS *Elaeocarpus angustifolius* | 0.001 | 0.001 | 0.001 | 0.001 | 0.001 | 0.001 | 0.001 | 0.001 |
| GT VS *Elaeocarpus angustifolius* | 0.001 | 0.001 | 0.001 | 0.001 | 0.001 | 0.001 | 0.001 | 0.001 |
| **Paired sample t-test** | | | | | | | | |
| **Group** | TG | | Cholesterol | | HDL | | LDL | |
|  | 0 day vs. 28^th^ days | | 0 day vs. 28^th^ days | | 0 day vs. 28^th^ days | | 0 day vs. 28^th^ days | |
| NWC | 0.557 | | 0.773 | | 0.044 | | 0.94 | |
| DWC | 0.001 | | 0.007 | | 0.009 | | 0.004 | |
| GT | 0.001 | | 0.003 | | 0.022 | | 0.001 | |
| *Elaeocarpus angustifolius* | 0.006 | | 0.034 | | 0.144 | | 0.040 | |

Group NWC, DWC, GT, and EA represent normal water control, diabetic water control rats, Gliclazide-treated, and *E. angustifolius-treated* rats, respectively. Data presented as mean±standard deviation (M±SD). Statistical comparison between groups was performed using one-way ANOVA and paired sample t-test.
