# Supplementary material for: Biochemical and antidiabetic properties of Elaeocarpus angustifolius Blume: In vitro, In vivo, and In silico insights
Source: PLoS One. 2026 Jun 8;21(6):e0349796. doi: 10.1371/journal.pone.0349796 (PMC13245756; doi:10.1371/journal.pone.0349796)
Supplement: S8 Table — (DOCX) [file pone.0349796.s013.docx]

S8 Table: Effect of *E.angustifolius* methanol extract on hepatic glycogen content.

| **Group** | **Glycogen 28^th^ days (mg/mL)** |
| --- | --- |
| NWC | 9.73 ± 0.001 |
| DWC | 1.39 ± 0.001 |
| GT | 12.82 ± 0.002 |
| *Elaeocarpus angustifolius* | 14.06 ± 0.002 |
| **One way ANOVA** | |
| NWC VS DWC | 0.001 |
| NWC VS GT | 0.016 |
| NWC VS *Elaeocarpus angustifolius* | 0.001 |
| DWC VS GT | 0.001 |
| DWC VS *Elaeocarpus angustifolius* | 0.001 |
| GT VS *Elaeocarpus angustifolius* | 0.001 |

Group NWC, DWC, GT, and EA represent normal water control, diabetic water control rats, Gliclazide treated, and *Elaeocarpus angustifolius* treated rat respectively. Data presented as mean±standard deviation (M±SD). Statistical comparison between groups was performed using one-way ANOVA.
