# Supplementary material for: Biochemical and antidiabetic properties of Elaeocarpus angustifolius Blume: In vitro, In vivo, and In silico insights
Source: PLoS One. 2026 Jun 8;21(6):e0349796. doi: 10.1371/journal.pone.0349796 (PMC13245756; doi:10.1371/journal.pone.0349796)
Supplement: Attachment — Submitted filename: revision.docx [file pone.0349796.s014.docx]

The manuscript presents a comprehensive *in vitro, in vivo,* and *in silico* investigation into the antidiabetic potential of *Elaeocarpus angustifolius* bark. The study is well-motivated, the experimental design is generally sound, and the findings are promising. However, several critical issues must be addressed to strengthen the manuscript's validity, clarity, and compliance with journal standards before it can be considered for publication.

**Major Revisions Required:**

1. The ethics statement on page 3 notes approval from the Bangladesh University of Health Sciences (BUHS) ERC. However, the in vivo study was conducted on rats in Nepal, while the approving body is in Bangladesh. Please clarify: Where exactly were the animals housed and the experiments performed? Provide the name and location of the animal facility.
2. Clarify the anesthesia/euthanasia method: The manuscript states anesthesia was performed using halothane. Please specify the method of euthanasia at the end of the experiment (e.g., overdose of anesthetic, cervical dislocation, etc.) as required by PLOS ONE's animal research guidelines (page 4 of the submission).
3. The Methods section (page 12) lists five groups but only names four (Group 1: NWC, Group 2: DWC, Group 3: GT, Group 5: Extract treated). There is a numbering error (Group 4 is missing). Please correct the group numbering and labeling throughout the manuscript and figures for consistency.
4. Justify the extract dosage: The dose of 1.25 g/kg is very high. Please provide a rationale for this specific dose (e.g., based on preliminary toxicity studies or literature). A brief discussion on the feasibility of translating such a high dose to human use should be added to the Discussion or Conclusion.
5. Clarify statistical tests: The legend for Figures 1 and 2 states a paired sample t-test was used for post-hoc comparison after ANOVA. This is unconventional. For comparing multiple groups, appropriate post-hoc tests (e.g., Tukey's, Bonferroni) should be used following ANOVA. Please specify the exact post-hoc test used and ensure it is appropriate for the experimental design.
6. For the *in vitro* enzyme inhibition assays (α-glucosidase and α-amylase), specify the positive control concentration range used alongside the samples. Currently, only the IC₅₀ of acarbose is given. This is necessary to validate the assay performance under the experimental conditions used for the extracts.
7. The Financial Disclosure section (page 2) states "The author(s) received no specific funding for this work." However, the Acknowledgements section (page 37) thanks the Asian Network of Research on Antidiabetic Plants (ANRAP) for "financial support." This is a direct contradiction. The authors must correct the Financial Disclosure statement to accurately reflect any and all sources of funding or material support received for this study, as per PLOS ONE policy.
8. Abstract: "significant progress in the discovery of antidiabetic drugs had been made" – change "had" to "has."
9. Page 23, Line 342: "The results (S4 Table) indicated..." – When referencing supporting information, briefly state the key finding in the main text (e.g., "The methanol extract exhibited the highest inhibition... (S4 Table)").
10. Discussion: The discussion could be strengthened by more directly comparing the potency (IC₅₀ values) of Corilagin's *in silico* binding with the observed *in vitro* activity of the ethyl acetate fraction. A sentence or two on this would better integrate the computational and experimental findings.
