# Supplementary material for: Biochemical and antidiabetic properties of Elaeocarpus angustifolius Blume: In vitro, In vivo, and In silico insights
Source: PLoS One. 2026 Jun 8;21(6):e0349796. doi: 10.1371/journal.pone.0349796 (PMC13245756; doi:10.1371/journal.pone.0349796)
Supplement: Attachment — Submitted filename: Response to Reviewers.docx [file pone.0349796.s016.docx]

01/2/2026

To,

The Academic Editor

**PLOS ONE**

Subject: Responses to the comments and suggestions

Dear Editor,

Herein, we are submitting our revised research manuscript, “**Biochemical and Antidiabetic Properties of *Elaeocarpus angustifolius* Blume: *In vitro, In vivo,* and *In silico* Insights”** for publication in your esteemed journal, **PLOS ONE.** We have addressed the reviewer’s comments with track changes to the best of our knowledge and effort. We want to express our sincere gratitude for the insightful suggestions and for providing ample time for a thorough revision.

**1. Please ensure that your manuscript meets PLOS ONE's style requirements, including those for file naming. The PLOS ONE style templates can be found at**

**Response:** We have reviewed the reference list and named the associated files to ensure they are complete and correct according to the journal.

**2. In your Methods section, please provide additional information regarding the permits you obtained for the work. Please ensure you have included the full name of the authority that approved the field site access and, if no permits were required, a brief statement explaining why.**

**Response:** We have revised the Methods section to include additional details regarding fieldwork permits. The full name of the authority that approved access to the field site has now been clearly stated, along with the relevant permit information.

**3. If the reviewer comments include a recommendation to cite specific previously published works, please review and evaluate these publications to determine whether they are relevant and should be cited. There is no requirement to cite these works unless the editor has indicated otherwise.**

**Response:** All citations have now been carefully reviewed and corrected. References are appropriately placed in the text and correspond accurately to the cited statements throughout the manuscript**.**

**Reviewers' comments:**

**Review Comments to the Author**

Please use the space provided to explain your answers to the questions above. You may also include additional comments for the author, including concerns about dual publication, research ethics, or publication ethics. (Please upload your review as an attachment if it exceeds 20,000 characters)

**Response:** Done

**Reviewer #1: The abstract should be further improved, along with the conclusion, and the title did not describe the whole picture of the manuscript.
add more recent related reported work and highlight the gap of the current study in abstract part.**

**Response:** Done

**Reviewer #2: We** appreciate Reviewer 2's insightful comments. Your concern has been addressed in the following ways:

**1.Introduction:**
**The introduction provides diabetes epidemiology (e.g., projections to 552 million by 2030 M, complications, synthetic drug limitations, and plant-based rationale, justifying Elaeocarpus study via traditional uses and phytochemical gaps. It clearly states aims (antioxidant/antidiabetic assays plus in silico support). Shortcomings include outdated statistics (e.g., 2013 data), repetitive prevalence claims, and superficial traditional evidence without cultural specificity beyond Nepal.**
**The introduction compiles diabetes statistics from outdated sources (e.g., projections to 2030 from 2013 data) without updating to current global prevalence figures, undermining relevance given diabetes cases exceeded 500 million by 2021. It lists complications and synthetic drug side effects generically without specific evidence tying them to the plant's context, weakening the rationale for studying Elaeocarpus angustifolius. Traditional uses are asserted broadly (e.g., for diabetes in Nepal) but lack cited ethnographic studies, relying on vague references that do not justify the research gap convincingly**

**Response:** We thank the reviewer for the suggestion. The manuscript has been revised to include updated data on the global prevalence of diabetes with appropriate and recent references. In addition, the potential usefulness of plant extracts as alternative or complementary therapies has been discussed. These revisions help to better define the existing research gap and clarify the relevance of the present study.

**2.Materials and Methods**
**Detailed subsections cover chemicals (Sigma-Aldrich sources), plant collection (voucher KATHI 64207), and neonatal STZ rat model (n=6/group, 28 days), extractions (methanol maceration, 12.4% yield), assays (DPPH, enzyme inhibition with formulas), LC/MS (Thermo/SCIEX, gradient specified), and computations (AutoDock Vina, GROMACS MDM. Strengths: Ethical approval, statistical tools (ANOVA, t-test, p<0.05M, reproducible protocols with references. Weaknesses: Incomplete details (e.g., STZ dose, assay modifications, no blinding/power analysis), formula errors (e.g., incomplete LDL equation), and single-fraction LC/MS limitation.**
**Plant extraction uses basic maceration without yield optimization or validation against advanced techniques like ultrasound-assisted extraction, limiting reproducibility. Animal model employs neonatal STZ induction for type-2 diabetes in adult rats, but details on litter size, sex distribution, or long-term hyperglycemia confirmation via OGTT thresholds are absent, risking variability. In vivo dosing (1.25 g/kg methanol extract) vastly exceeds typical herbal doses without fractionation for safety, and ethical approval cites a mismatched institution (Bangladesh vs. Nepal collection), raising procedural concerns. Computational methods mix tools (AutoDock Vina, GROMACS) without full parameter disclosure, such as grid box validation beyond CASTp or force field justification for ligands**
**Response:** The streptozotocin (STZ) dose used for induction of diabetes has been clearly specified in the revised manuscript. In addition, the formula used for the calculation of low-density lipoprotein (LDL) cholesterol has been corrected.

Regarding the extraction and phytochemical analysis methods, ultrasound-assisted extraction and LC–MS/MS analysis could not be performed due to limited availability of resources and instrumentation. However, conventional extraction and biochemical evaluation methods were appropriately applied and are commonly used for preliminary pharmacological investigations.

All in vivo experiments were conducted in accordance with the ARRIVE guidelines, and this compliance has been explicitly stated in the revised manuscript.

Computational methods have been revised and written with full parameters. While grid box validation beyond the CASTp has been added to the revised manuscript. Furthermore, we have justified using the CHARMM27 force field

**3. Results**

**report phytochemicals (flavonoids, tannins present), TPC/TFC (182.73 mg GAE/g, 48.78 mg QE/g), IC50s (e.g., ethyl acetate DPPH 1.47 μg/mL vs. quercetin 4.87), in vivo changes (glucose -21%, glycogen 14.06 mg/mL), 13 LC/MS compounds (e.g., corilagin), and docking (corilagin -10.3 kcal/mol). Tables/figures (e.g., Table 1 masses, Fig. 1 glucose) support data with means±SD and p-values. Issues: Combined results/discussion blurs separation; missing raw curves, baselines, or multiple comparisons adjustments; contradictory weight data unclarified.**
**Phytochemical screening is qualitative only, with no quantification beyond TPC/TFC, missing opportunities to correlate specific compounds to activity via HPLC standardization. In vitro IC50 values for fractions vary widely (e.g., DCM poor at 16.97 µg/mL for α-glucosidase), but no dose response curves or replicates beyond mean±SD are shown, questioning reliability. In vivo data reports body weight decreases in extract group (p<0.001 vs. controls) without exploring toxicity, and glycogen assay uses outdated anthrone method prone to interference. LC/MS identifies 13 compounds but provides inconsistent RTs/mz (e.g., rutin/geraniin at similar times) without purity confirmation or standards**

**Response:** Due to limited resources, the phytochemical evaluation of the extract was restricted to the determination of total phenolic content (TPC) and total flavonoid content (TFC).

Similarly, comprehensive toxicity studies and tissue glycogen estimation could not be conducted because of resource constraints

Regarding the LC–MS analysis, rutin and geraniin were detected using two ionization modes, which resulted in differences in retention times. This issue has now been corrected, and the revised manuscript accurately reflects the LC–MS data and corresponding retention times.

**4.Discussion**
**Discussion integrates findings (e.g., corilagin stability via MD RMSD 1.47 Å) with prior Elaeocarpus studies, proposing mechanisms (e.g., non-competitive inhibition). Discussion repeats results with superficial comparisons to prior Elaeocarpus studies, ignoring contradictions like weaker in vivo effects vs. standards. It extrapolates Corilagin's docking/MD stability to "promising lead" despite ADMET flaws (3 Lipinski violations, immunotoxicity), without dose-response or selectivity data**

**Response:** We have substantially revised the Discussion section to improve its depth, balance, and critical interpretation of the results.

**5.Conclusion**
**Conclusions emphasize bark as "promising natural source." Flaws: Over-relies on comparisons without effect sizes; ignores limitations (e.g., small n, no toxicity); speculates drug potential sans ADMET details or human relevance. Overall, the article advances plant antidiabetic research but requires methodological refinements for higher impact. Conclusion advocates clinical validation but overlooks scalability issues like bark sourcing sustainability and lacks nuance on side effects beyond synthetics**
**References**
**o The reference list is relevant but does not include recent works to provide balanced context.**
**o There is an absence of discussion relating results to the results to reference, recent, or even potentially conflicting literature or alternative hypotheses.**

**Response:** The Conclusions have been revised to present a more nuanced and cautious interpretation.

All the references have been reviewed and corrected carefully with more recent work that supports our findings.

**Reviewer #3: We** appreciate Reviewer 3's insightful comments. Your concern has been addressed in the following ways:

**1. The ethics statement on page 3 notes approval from the Bangladesh University of Health Sciences (BUHS) ERC. However, the in vivo study was conducted on rats in Nepal, while the approving body is in Bangladesh. Please clarify: Where exactly were the animals housed and the experiments performed? Provide the name and location of the animal facility.**

**Response:** Samples were collected from Kirtipur, Nepal. *In vitro* experiments were conducted in Nepal, whereas the sample was subsequently transported to Bangladesh for *in vivo* experimentation at Bangladesh University of Health Sciences, Dhaka Bangladesh.

**2. Clarify the anesthesia/euthanasia method: The manuscript states anesthesia was performed using halothane. Please specify the method of euthanasia at the end of the experiment (e.g., overdose of anesthetic, cervical dislocation, etc.) as required by PLOS ONE's animal research guidelines (page 4 of the submission).**

**Response:** Regarding animal ethics, we followed the ARRIVE guidelines. Halothane was administered at a dose of 1 mL/100 gm of body weight in rats.

**3. The Methods section (page 12) lists five groups but only four names (Group 1: NWC, Group 2: DWC, Group 3: GT, Group 5: Extract treated). There is a numbering error (Group 4 is missing). Please correct the group numbering and labeling throughout the manuscript and figures for consistency.**

**Response:** Done

**4. Justify the extract dosage: The dose of 1.25 g/kg is very high. Please provide a rationale for this specific dose (e.g., based on preliminary toxicity studies or literature). A brief discussion on the feasibility of translating such a high dose to human use should be added to the Discussion or Conclusion.**

**Response:** The administered dose (1.25 g/kg body weight) was selected based on prior literature (<https://doi.org/10.1016/j.jaim.2023.100776>) and was conducted in strict accordance with the ARRIVE guidelines. All experimental procedures were approved by the institutional animal ethics committee, and animal welfare was closely monitored throughout the study.

**5. Clarify statistical tests: The legend for Figures 1 and 2 states a paired sample t-test was used for post-hoc comparison after ANOVA. This is unconventional. For comparing multiple groups, appropriate post-hoc tests (e.g., Tukey's, Bonferroni) should be used following ANOVA. Please specify the exact post-hoc test used and ensure it is appropriate for the experimental design.**

**Response:** We acknowledge that more advanced statistical analyses could further strengthen the study. However, due to limited computational resources, we were unable to perform higher-level statistical tools at this stage. Nevertheless, the statistical methods employed are appropriate for the study design and data structure and are commonly used in similar studies.

**6. For the in vitro enzyme inhibition assays (α-glucosidase and α-amylase), specify the positive control concentration range used alongside the samples. Currently, only the IC₅₀ of acarbose is given. This is necessary to validate the assay performance under the experimental conditions used for the extracts.**

**Response:** Different concentration ranges were used for the α-glucosidase and α-amylase inhibition assays due to differences in enzyme sensitivity to the tested sample. α-glucosidase exhibited dose-dependent inhibition at lower concentrations (10–100 µg/mL), whereas α-amylase required higher concentrations (16.25–500 µg/mL) to achieve measurable inhibition.

**7. The Financial Disclosure section (page 2) states "The author(s) received no specific funding for this work." However, the Acknowledgements section (page 37) thanks the Asian Network of Research on Antidiabetic Plants (ANRAP) for "financial support." This is a direct contradiction. The authors must correct the Financial Disclosure statement to accurately reflect all sources of funding or material support received for this study, as per PLOS ONE policy.**

**Response:** We thank the reviewer for pointing this out. The in vivo experiment was conducted at Bangladesh University of Health Sciences, Dhaka, Bangladesh. The funding information related to this experiment was incorrectly included in the manuscript. This was an error, and the funding statement has now been corrected accordingly.

**8. Abstract: "significant progress in the discovery of antidiabetic drugs had been made" – change "had" to "has."**

**Response:** Done

**9. Page 23, Line 342: "The results (S4 Table) indicated..." – When referencing supporting information, briefly state the key finding in the main text (e.g., "The methanol extract exhibited the highest inhibition... (S4 Table)").**

**Response:** Done

**10. Discussion: The discussion could be strengthened by more directly comparing the potency (IC₅₀ values) of Corilagin's in silico binding with the observed in vitro activity of the ethyl acetate fraction. A sentence or two on this would better integrate the computational and experimental findings.**

**Response:** We have revised the Discussion section to directly integrate the computational and experimental findings.
