# Supplementary material for: Biochemical and antidiabetic properties of Elaeocarpus angustifolius Blume: In vitro, In vivo, and In silico insights
Source: PLoS One. 2026 Jun 8;21(6):e0349796. doi: 10.1371/journal.pone.0349796 (PMC13245756; doi:10.1371/journal.pone.0349796)
Supplement: Peer review history

# Peer review: Decision Letter 0

**DOI:** 10.1371/journal.pone.0349796.r001

**Authors:** Jorddy Neves Cruz

26 Dec 2025

PONE-D-25-60521

Biochemical and Antidiabetic Properties of Elaeocarpus angustifolius Blume: In vitro, In vivo, and In silico Insights

PLOS One

Dear Dr. Adhikari,

Thank you for submitting your manuscript to PLOS ONE. After careful consideration, we feel that it has merit but does not fully meet PLOS ONE’s publication criteria as it currently stands. Therefore, we invite you to submit a revised version of the manuscript that addresses the points raised during the review process.

Please submit your revised manuscript by Feb 09 2026 11:59PM. If you will need more time than this to complete your revisions, please reply to this message or contact the journal office at plosone@plos.org. When you're ready to submit your revision, log on to https://www.editorialmanager.com/pone/ and select the 'Submissions Needing Revision' folder to locate your manuscript file.

Please include the following items when submitting your revised manuscript:

- A letter that responds to each point raised by the academic editor and reviewer(s). You should upload this letter as a separate file labeled 'Response to Reviewers'.
- A marked-up copy of your manuscript that highlights changes made to the original version. You should upload this as a separate file labeled 'Revised Manuscript with Track Changes'.
- An unmarked version of your revised paper without tracked changes. You should upload this as a separate file labeled 'Manuscript'.

If you would like to make changes to your financial disclosure, please include your updated statement in your cover letter. Guidelines for resubmitting your figure files are available below the reviewer comments at the end of this letter.

If applicable, we recommend that you deposit your laboratory protocols in protocols.io to enhance the reproducibility of your results. Protocols.io assigns your protocol its own identifier (DOI) so that it can be cited independently in the future. For instructions see: https://journals.plos.org/plosone/s/submission-guidelines#loc-laboratory-protocols. Additionally, PLOS ONE offers an option for publishing peer-reviewed Lab Protocol articles, which describe protocols hosted on protocols.io. Read more information on sharing protocols at https://plos.org/protocols?utm_medium=editorial-email&utm_source=authorletters&utm_campaign=protocols.

We look forward to receiving your revised manuscript.

Kind regards,

Jorddy Neves Cruz

Academic Editor

PLOS One

Journal Requirements:

When submitting your revision, we need you to address these additional requirements.

1. Please ensure that your manuscript meets PLOS ONE's style requirements, including those for file naming. The PLOS ONE style templates can be found at https://journals.plos.org/plosone/s/file?id=wjVg/PLOSOne_formatting_sample_main_body.pdf and https://journals.plos.org/plosone/s/file?id=ba62/PLOSOne_formatting_sample_title_authors_affiliations.pdf

2. In your Methods section, please provide additional information regarding the permits you obtained for the work. Please ensure you have included the full name of the authority that approved the field site access and, if no permits were required, a brief statement explaining why.

3. If the reviewer comments include a recommendation to cite specific previously published works, please review and evaluate these publications to determine whether they are relevant and should be cited. There is no requirement to cite these works unless the editor has indicated otherwise.

[Note: HTML markup is below. Please do not edit.]

Reviewers' comments:

Reviewer's Responses to Questions

**Comments to the Author**

1. Is the manuscript technically sound, and do the data support the conclusions?

The manuscript must describe a technically sound piece of scientific research with data that supports the conclusions. Experiments must have been conducted rigorously, with appropriate controls, replication, and sample sizes. The conclusions must be drawn appropriately based on the data presented.

Reviewer #1: Yes

Reviewer #2: Yes

Reviewer #3: Yes

**********

2. Has the statistical analysis been performed appropriately and rigorously?

Reviewer #1: Yes

Reviewer #2: Yes

Reviewer #3: Yes

**********

3. Have the authors made all data underlying the findings in their manuscript fully available?

The PLOS Data policy requires authors to make all data underlying the findings described in their manuscript fully available without restriction, with rare exception (please refer to the Data Availability Statement in the manuscript PDF file). The data should be provided as part of the manuscript or its supporting information, or deposited to a public repository. For example, in addition to summary statistics, the data points behind means, medians and variance measures should be available. If there are restrictions on publicly sharing data—e.g. participant privacy or use of data from a third party—those must be specified.

Reviewer #1: Yes

Reviewer #2: Yes

Reviewer #3: Yes

**********

4. Is the manuscript presented in an intelligible fashion and written in standard English?

PLOS ONE does not copyedit accepted manuscripts, so the language in submitted articles must be clear, correct, and unambiguous. Any typographical or grammatical errors should be corrected at revision, so please note any specific errors here.

Reviewer #1: Yes

Reviewer #2: Yes

Reviewer #3: Yes

**********

5. Review Comments to the Author

Please use the space provided to explain your answers to the questions above. You may also include additional comments for the author, including concerns about dual publication, research ethics, or publication ethics. (Please upload your review as an attachment if it exceeds 20,000 characters)

Reviewer #1: The abstract should be further improved, along with the conclusion, and the title did not describe the whole picture of the manuscript.

add more recent related reported work and highlight the gap of the current study in abstract part

Reviewer #2: Article review

Title: Biochemical and Antidiabetic Properties of Elaeocarpus angustifolius Blume: In vitro, In vivo, and In silico Insights.

The manuscript is skilfully written. The article evaluates the antidiabetic potential of Elaeocarpus angustifolius bark extract through phytochemical, in vitro, in vivo, and computational analyses, structured across standard scientific sections with varying strengths in clarity and depth. While the study provides valuable insights and a robust dataset, several weaknesses and limitations can be discerned in its different components. The results can be presented in a more suitable manner. In summary, certain remarks ought to be taken into account.

Title and abstract

The title adequately reflects the study content. Please add from Kirtipur, Kathmandu .

The abstract concisely outlines objectives, methods, key results (e.g., IC50 values for α-glucosidase 0.79 μg/mL, glucose reduction 21%, corilagin docking ]10.3 kcal/mol), and implications for drug development, effectively highlighting antioxidant/antidiabetic activities. Strengths include quantitative highlights and logical flow from in vitro to in silico data. The abstract provides a dense summary of methods and results but lacks precision in quantifying key outcomes, such as exact reductions in glucose levels or statistical significance beyond p<0.01 mentions. It overstates findings by claiming "potent" inhibition without contextualizing IC50 values against standards like acarbose, potentially misleading readers on comparative efficacy. Additionally, it prematurely highlights Corilagin as the "most active compound" without briefly noting limitations like in vivo dose translation.

1. Introduction:

The introduction provides diabetes epidemiology (e.g., projections to 552 million by 2030 M, complications, synthetic drug limitations, and plant-based rationale, justifying Elaeocarpus study via traditional uses and phytochemical gaps. It clearly states aims (antioxidant/antidiabetic assays plus in silico support). Shortcomings include outdated statistics (e.g., 2013 data), repetitive prevalence claims, and superficial traditional evidence without cultural specificity beyond Nepal.

The introduction compiles diabetes statistics from outdated sources (e.g., projections to 2030 from 2013 data) without updating to current global prevalence figures, undermining relevance given diabetes cases exceeded 500 million by 2021. It lists complications and synthetic drug side effects generically without specific evidence tying them to the plant's context, weakening the rationale for studying Elaeocarpus angustifolius. Traditional uses are asserted broadly (e.g., for diabetes in Nepal) but lack cited ethnographic studies, relying on vague references that do not justify the research gap convincingly

2. Materials and Methods

Detailed subsections cover chemicals (Sigma-Aldrich sources), plant collection (voucher KATHI 64207), and neonatal STZ rat model (n=6/group, 28 days), extractions (methanol maceration, 12.4% yield), assays (DPPH, enzyme inhibition with formulas), LC/MS (Thermo/SCIEX, gradient specified), and computations (AutoDock Vina, GROMACS MDM. Strengths: Ethical approval, statistical tools (ANOVA, t-test, p<0.05M, reproducible protocols with references. Weaknesses: Incomplete details (e.g., STZ dose, assay modifications, no blinding/power analysis), formula errors (e.g., incomplete LDL equation), and single-fraction LC/MS limitation.

Plant extraction uses basic maceration without yield optimization or validation against advanced techniques like ultrasound-assisted extraction, limiting reproducibility. Animal model employs neonatal STZ induction for type-2 diabetes in adult rats, but details on litter size, sex distribution, or long-term hyperglycemia confirmation via OGTT thresholds are absent, risking variability. In vivo dosing (1.25 g/kg methanol extract) vastly exceeds typical herbal doses without fractionation for safety, and ethical approval cites a mismatched institution (Bangladesh vs. Nepal collection), raising procedural concerns. Computational methods mix tools (AutoDock Vina, GROMACS) without full parameter disclosure, such as grid box validation beyond CASTp or force field justification for ligands

3. Results

Results report phytochemicals (flavonoids, tannins present), TPC/TFC (182.73 mg GAE/g, 48.78 mg QE/g), IC50s (e.g., ethyl acetate DPPH 1.47 μg/mL vs. quercetin 4.87), in vivo changes (glucose -21%, glycogen 14.06 mg/mL), 13 LC/MS compounds (e.g., corilagin), and docking (corilagin -10.3 kcal/mol). Tables/figures (e.g., Table 1 masses, Fig. 1 glucose) support data with means±SD and p-values. Issues: Combined results/discussion blurs separation; missing raw curves, baselines, or multiple comparisons adjustments; contradictory weight data unclarified.

Phytochemical screening is qualitative only, with no quantification beyond TPC/TFC, missing opportunities to correlate specific compounds to activity via HPLC standardization. In vitro IC50 values for fractions vary widely (e.g., DCM poor at 16.97 µg/mL for α-glucosidase), but no dose response curves or replicates beyond mean±SD are shown, questioning reliability. In vivo data reports body weight decreases in extract group (p<0.001 vs. controls) without exploring toxicity, and glycogen assay uses outdated anthrone method prone to interference. LC/MS identifies 13 compounds but provides inconsistent RTs/mz (e.g., rutin/geraniin at similar times) without purity confirmation or standards

4. Discussion

Discussion integrates findings (e.g., corilagin stability via MD RMSD 1.47 Å) with prior Elaeocarpus studies, proposing mechanisms (e.g., non-competitive inhibition). Discussion repeats results with superficial comparisons to prior Elaeocarpus studies, ignoring contradictions like weaker in vivo effects vs. standards. It extrapolates Corilagin's docking/MD stability to "promising lead" despite ADMET flaws (3 Lipinski violations, immunotoxicity), without dose-response or selectivity data

5. Conclusion

Conclusions emphasize bark as "promising natural source." Flaws: Over-relies on comparisons without effect sizes; ignores limitations (e.g., small n, no toxicity); speculates drug potential sans ADMET details or human relevance. Overall, the article advances plant antidiabetic research but requires methodological refinements for higher impact. Conclusion advocates clinical validation but overlooks scalability issues like bark sourcing sustainability and lacks nuance on side effects beyond synthetics

References

o The reference list is relevant but does not include recent works to provide balanced context.

o There is an absence of discussion relating results to the results to reference, recent, or even potentially conflicting literature or alternative hypotheses.

Reviewer #3: The manuscript presents a comprehensive in vitro, in vivo, and in silico investigation into the antidiabetic potential of Elaeocarpus angustifolius bark. The study is well-motivated, the experimental design is generally sound, and the findings are promising. However, several critical issues must be addressed to strengthen the manuscript's validity, clarity, and compliance with journal standards before it can be considered for publication.

Major Revisions Required:

1. The ethics statement on page 3 notes approval from the Bangladesh University of Health Sciences (BUHS) ERC. However, the in vivo study was conducted on rats in Nepal, while the approving body is in Bangladesh. Please clarify: Where exactly were the animals housed and the experiments performed? Provide the name and location of the animal facility.

2. Clarify the anesthesia/euthanasia method: The manuscript states anesthesia was performed using halothane. Please specify the method of euthanasia at the end of the experiment (e.g., overdose of anesthetic, cervical dislocation, etc.) as required by PLOS ONE's animal research guidelines (page 4 of the submission).

3. The Methods section (page 12) lists five groups but only names four (Group 1: NWC, Group 2: DWC, Group 3: GT, Group 5: Extract treated). There is a numbering error (Group 4 is missing). Please correct the group numbering and labeling throughout the manuscript and figures for consistency.

4. Justify the extract dosage: The dose of 1.25 g/kg is very high. Please provide a rationale for this specific dose (e.g., based on preliminary toxicity studies or literature). A brief discussion on the feasibility of translating such a high dose to human use should be added to the Discussion or Conclusion.

5. Clarify statistical tests: The legend for Figures 1 and 2 states a paired sample t-test was used for post-hoc comparison after ANOVA. This is unconventional. For comparing multiple groups, appropriate post-hoc tests (e.g., Tukey's, Bonferroni) should be used following ANOVA. Please specify the exact post-hoc test used and ensure it is appropriate for the experimental design.

6. For the in vitro enzyme inhibition assays (α-glucosidase and α-amylase), specify the positive control concentration range used alongside the samples. Currently, only the IC₅₀ of acarbose is given. This is necessary to validate the assay performance under the experimental conditions used for the extracts.

7. The Financial Disclosure section (page 2) states "The author(s) received no specific funding for this work." However, the Acknowledgements section (page 37) thanks the Asian Network of Research on Antidiabetic Plants (ANRAP) for "financial support." This is a direct contradiction. The authors must correct the Financial Disclosure statement to accurately reflect any and all sources of funding or material support received for this study, as per PLOS ONE policy.

8. Abstract: "significant progress in the discovery of antidiabetic drugs had been made" – change "had" to "has."

9. Page 23, Line 342: "The results (S4 Table) indicated..." – When referencing supporting information, briefly state the key finding in the main text (e.g., "The methanol extract exhibited the highest inhibition... (S4 Table)").

10. Discussion: The discussion could be strengthened by more directly comparing the potency (IC₅₀ values) of Corilagin's in silico binding with the observed in vitro activity of the ethyl acetate fraction. A sentence or two on this would better integrate the computational and experimental findings.

**********

6. PLOS authors have the option to publish the peer review history of their article (what does this mean?). If published, this will include your full peer review and any attached files.

If you choose “no”, your identity will remain anonymous but your review may still be made public.

**Do you want your identity to be public for this peer review?** For information about this choice, including consent withdrawal, please see our Privacy Policy.

Reviewer #1: No

Reviewer #2: No

Reviewer #3: **Yes:**Amal El Rherabi

**********

[NOTE: If reviewer comments were submitted as an attachment file, they will be attached to this email and accessible via the submission site. Please log into your account, locate the manuscript record, and check for the action link "View Attachments". If this link does not appear, there are no attachment files.]

To ensure your figures meet our technical requirements, please review our figure guidelines: https://journals.plos.org/plosone/s/figures

You may also use PLOS’s free figure tool, NAAS, to help you prepare publication quality figures: https://journals.plos.org/plosone/s/figures#loc-tools-for-figure-preparation.

NAAS will assess whether your figures meet our technical requirements by comparing each figure against our figure specifications.

Attachment Submitted filename: revision.docx

# Author comment: Author response to Decision Letter 1

**DOI:** 10.1371/journal.pone.0349796.r002

2 Feb 2026

1. Please ensure that your manuscript meets PLOS ONE's style requirements, including those for file naming. The PLOS ONE style templates can be found at

Response: We have reviewed the reference list and named the associated files to ensure they are complete and correct according to the journal.

2. In your Methods section, please provide additional information regarding the permits you obtained for the work. Please ensure you have included the full name of the authority that approved the field site access and, if no permits were required, a brief statement explaining why.

Response: We have revised the Methods section to include additional details regarding fieldwork permits. The full name of the authority that approved access to the field site has now been clearly stated, along with the relevant permit information.

3. If the reviewer comments include a recommendation to cite specific previously published works, please review and evaluate these publications to determine whether they are relevant and should be cited. There is no requirement to cite these works unless the editor has indicated otherwise.

Response: All citations have now been carefully reviewed and corrected. References are appropriately placed in the text and correspond accurately to the cited statements throughout the manuscript.

Reviewers' comments:

Review Comments to the Author

Please use the space provided to explain your answers to the questions above. You may also include additional comments for the author, including concerns about dual publication, research ethics, or publication ethics. (Please upload your review as an attachment if it exceeds 20,000 characters)

Response: Done

Reviewer #1: The abstract should be further improved, along with the conclusion, and the title did not describe the whole picture of the manuscript.

add more recent related reported work and highlight the gap of the current study in abstract part.

Response: Done

Reviewer #2: We appreciate Reviewer 2's insightful comments. Your concern has been addressed in the following ways:

1.Introduction:

The introduction provides diabetes epidemiology (e.g., projections to 552 million by 2030 M, complications, synthetic drug limitations, and plant-based rationale, justifying Elaeocarpus study via traditional uses and phytochemical gaps. It clearly states aims (antioxidant/antidiabetic assays plus in silico support). Shortcomings include outdated statistics (e.g., 2013 data), repetitive prevalence claims, and superficial traditional evidence without cultural specificity beyond Nepal.

The introduction compiles diabetes statistics from outdated sources (e.g., projections to 2030 from 2013 data) without updating to current global prevalence figures, undermining relevance given diabetes cases exceeded 500 million by 2021. It lists complications and synthetic drug side effects generically without specific evidence tying them to the plant's context, weakening the rationale for studying Elaeocarpus angustifolius. Traditional uses are asserted broadly (e.g., for diabetes in Nepal) but lack cited ethnographic studies, relying on vague references that do not justify the research gap convincingly

Response: We thank the reviewer for the suggestion. The manuscript has been revised to include updated data on the global prevalence of diabetes with appropriate and recent references. In addition, the potential usefulness of plant extracts as alternative or complementary therapies has been discussed. These revisions help to better define the existing research gap and clarify the relevance of the present study.

2.Materials and Methods

Detailed subsections cover chemicals (Sigma-Aldrich sources), plant collection (voucher KATHI 64207), and neonatal STZ rat model (n=6/group, 28 days), extractions (methanol maceration, 12.4% yield), assays (DPPH, enzyme inhibition with formulas), LC/MS (Thermo/SCIEX, gradient specified), and computations (AutoDock Vina, GROMACS MDM. Strengths: Ethical approval, statistical tools (ANOVA, t-test, p<0.05M, reproducible protocols with references. Weaknesses: Incomplete details (e.g., STZ dose, assay modifications, no blinding/power analysis), formula errors (e.g., incomplete LDL equation), and single-fraction LC/MS limitation.

Plant extraction uses basic maceration without yield optimization or validation against advanced techniques like ultrasound-assisted extraction, limiting reproducibility. Animal model employs neonatal STZ induction for type-2 diabetes in adult rats, but details on litter size, sex distribution, or long-term hyperglycemia confirmation via OGTT thresholds are absent, risking variability. In vivo dosing (1.25 g/kg methanol extract) vastly exceeds typical herbal doses without fractionation for safety, and ethical approval cites a mismatched institution (Bangladesh vs. Nepal collection), raising procedural concerns. Computational methods mix tools (AutoDock Vina, GROMACS) without full parameter disclosure, such as grid box validation beyond CASTp or force field justification for ligands

Response: The streptozotocin (STZ) dose used for induction of diabetes has been clearly specified in the revised manuscript. In addition, the formula used for the calculation of low-density lipoprotein (LDL) cholesterol has been corrected.

Regarding the extraction and phytochemical analysis methods, ultrasound-assisted extraction and LC–MS/MS analysis could not be performed due to limited availability of resources and instrumentation. However, conventional extraction and biochemical evaluation methods were appropriately applied and are commonly used for preliminary pharmacological investigations.

All in vivo experiments were conducted in accordance with the ARRIVE guidelines, and this compliance has been explicitly stated in the revised manuscript.

Computational methods have been revised and written with full parameters. While grid box validation beyond the CASTp has been added to the revised manuscript. Furthermore, we have justified using the CHARMM27 force field

3. Results

report phytochemicals (flavonoids, tannins present), TPC/TFC (182.73 mg GAE/g, 48.78 mg QE/g), IC50s (e.g., ethyl acetate DPPH 1.47 μg/mL vs. quercetin 4.87), in vivo changes (glucose -21%, glycogen 14.06 mg/mL), 13 LC/MS compounds (e.g., corilagin), and docking (corilagin -10.3 kcal/mol). Tables/figures (e.g., Table 1 masses, Fig. 1 glucose) support data with means±SD and p-values. Issues: Combined results/discussion blurs separation; missing raw curves, baselines, or multiple comparisons adjustments; contradictory weight data unclarified.

Phytochemical screening is qualitative only, with no quantification beyond TPC/TFC, missing opportunities to correlate specific compounds to activity via HPLC standardization. In vitro IC50 values for fractions vary widely (e.g., DCM poor at 16.97 µg/mL for α-glucosidase), but no dose response curves or replicates beyond mean±SD are shown, questioning reliability. In vivo data reports body weight decreases in extract group (p<0.001 vs. controls) without exploring toxicity, and glycogen assay uses outdated anthrone method prone to interference. LC/MS identifies 13 compounds but provides inconsistent RTs/mz (e.g., rutin/geraniin at similar times) without purity confirmation or standards

Response: Due to limited resources, the phytochemical evaluation of the extract was restricted to the determination of total phenolic content (TPC) and total flavonoid content (TFC).

Similarly, comprehensive toxicity studies and tissue glycogen estimation could not be conducted because of resource constraints

Regarding the LC–MS analysis, rutin and geraniin were detected using two ionization modes, which resulted in differences in retention times. This issue has now been corrected, and the revised manuscript accurately reflects the LC–MS data and corresponding retention times.

4.Discussion

Discussion integrates findings (e.g., corilagin stability via MD RMSD 1.47 Å) with prior Elaeocarpus studies, proposing mechanisms (e.g., non-competitive inhibition). Discussion repeats results with superficial comparisons to prior Elaeocarpus studies, ignoring contradictions like weaker in vivo effects vs. standards. It extrapolates Corilagin's docking/MD stability to "promising lead" despite ADMET flaws (3 Lipinski violations, immunotoxicity), without dose-response or selectivity data

Response: We have substantially revised the Discussion section to improve its depth, balance, and critical interpretation of the results.

5.Conclusion

Conclusions emphasize bark as "promising natural source." Flaws: Over-relies on comparisons without effect sizes; ignores limitations (e.g., small n, no toxicity); speculates drug potential sans ADMET details or human relevance. Overall, the article advances plant antidiabetic research but requires methodological refinements for higher impact. Conclusion advocates clinical validation but overlooks scalability issues like bark sourcing sustainability and lacks nuance on side effects beyond synthetics

References

o The reference list is relevant but does not include recent works to provide balanced context.

o There is an absence of discussion relating results to the results to reference, recent, or even potentially conflicting literature or alternative hypotheses.

Response: The Conclusions have been revised to present a more nuanced and cautious interpretation.

All the references have been reviewed and corrected carefully with more recent work that supports our findings.

Reviewer #3: We appreciate Reviewer 3's insightful comments. Your concern has been addressed in the following ways:

1. The ethics statement on page 3 notes approval from the Bangladesh University of Health Sciences (BUHS) ERC. However, the in vivo study was conducted on rats in Nepal, while the approving body is in Bangladesh. Please clarify: Where exactly were the animals housed and the experiments performed? Provide the name and location of the animal facility.

Response: Samples were collected from Kirtipur, Nepal. In vitro experiments were conducted in Nepal, whereas the sample was subsequently transported to Bangladesh for in vivo experimentation at Bangladesh University of Health Sciences, Dhaka Bangladesh.

2. Clarify the anesthesia/euthanasia method: The manuscript states anesthesia was performed using halothane. Please specify the method of euthanasia at the end of the experiment (e.g., overdose of anesthetic, cervical dislocation, etc.) as required by PLOS ONE's animal research guidelines (page 4 of the submission).

Response: Regarding animal ethics, we followed the ARRIVE guidelines. Halothane was administered at a dose of 1 mL/100 gm of body weight in rats.

3. The Methods section (page 12) lists five groups but only four names (Group 1: NWC, Group 2: DWC, Group 3: GT, Group 5: Extract treated). There is a numbering error (Group 4 is missing). Please correct the group numbering and labeling throughout the manuscript and figures for consistency.

Response: Done

4. Justify the extract dosage: The dose of 1.25 g/kg is very high. Please provide a rationale for this specific dose (e.g., based on preliminary toxicity studies or literature). A brief discussion on the feasibility of translating such a high dose to human use should be added to the Discussion or Conclusion.

Response: The administered dose (1.25 g/kg body weight) was selected based on prior literature (https://doi.org/10.1016/j.jaim.2023.100776) and was conducted in strict accordance with the ARRIVE guidelines. All experimental procedures were approved by the institutional animal ethics committee, and animal welfare was closely monitored throughout the study.

5. Clarify statistical tests: The legend for Figures 1 and 2 states a paired sample t-test was used for post-hoc comparison after ANOVA. This is unconventional. For comparing multiple groups, appropriate post-hoc tests (e.g., Tukey's, Bonferroni) should be used following ANOVA. Please specify the exact post-hoc test used and ensure it is appropriate for the experimental design.

Response: We acknowledge that more advanced statistical analyses could further strengthen the study. However, due to limited computational resources, we were unable to perform higher-level statistical tools at this stage. Nevertheless, the statistical methods employed are appropriate for the study design and data structure and are commonly used in similar studies.

6. For the in vitro enzyme inhibition assays (α-glucosidase and α-amylase), specify the positive control concentration range used alongside the samples. Currently, only the IC₅₀ of acarbose is given. This is necessary to validate the assay performance under the experimental conditions used for the extracts.

Response: Different concentration ranges were used for the α-glucosidase and α-amylase inhibition assays due to differences in enzyme sensitivity to the tested sample. α-glucosidase exhibited dose-dependent inhibition at lower concentrations (10–100 µg/mL), whereas α-amylase required higher concentrations (16.25–500 µg/mL) to achieve measurable inhibition.

7. The Financial Disclosure section (page 2) states "The author(s) received no specific funding for this work." However, the Acknowledgements section (page 37) thanks the Asian Network of Research on Antidiabetic Plants (ANRAP) for "financial support." This is a direct contradiction. The authors must correct the Financial Disclosure statement to accurately reflect all sources of funding or material support received for this study, as per PLOS ONE policy.

Response: We thank the reviewer for pointing this out. The in vivo experiment was conducted at Bangladesh University of Health Sciences, Dhaka, Bangladesh. The funding information related to this experiment was incorrectly included in the manuscript. This was an error, and the funding statement has now been corrected accordingly.

8. Abstract: "significant progress in the discovery of antidiabetic drugs had been made" – change "had" to "has."

Response: Done

9. Page 23, Line 342: "The results (S4 Table) indicated..." – When referencing supporting information, briefly state the key finding in the main text (e.g., "The methanol extract exhibited the highest inhibition... (S4 Table)").

Response: Done

10. Discussion: The discussion could be strengthened by more directly comparing the potency (IC₅₀ values) of Corilagin's in silico binding with the observed in vitro activity of the ethyl acetate fraction. A sentence or two on this would better integrate the computational and experimental findings.

Response: We have revised the Discussion section to directly integrate the computational and experimental findings.

Attachment Submitted filename: Response to Reviewers.docx

# Peer review: Decision Letter 1

**DOI:** 10.1371/journal.pone.0349796.r003

**Authors:** Jorddy Neves Cruz

6 May 2026

Biochemical and Antidiabetic Properties of Elaeocarpus angustifolius Blume: In vitro, In vivo, and In silico Insights

PONE-D-25-60521R1

Dear Dr. Adhikari,

We’re pleased to inform you that your manuscript has been judged scientifically suitable for publication and will be formally accepted for publication once it meets all outstanding technical requirements.

Within one week, you’ll receive an e-mail detailing the required amendments. When these have been addressed, you’ll receive a formal acceptance letter and your manuscript will be scheduled for publication.

An invoice will be generated when your article is formally accepted. Please note, if your institution has a publishing partnership with PLOS and your article meets the relevant criteria, all or part of your publication costs will be covered. Please make sure your user information is up-to-date by logging into Editorial Manager at Editorial Manager® and clicking the ‘Update My Information' link at the top of the page. For questions related to billing, please contact billing support.

If your institution or institutions have a press office, please notify them about your upcoming paper to help maximize its impact. If they’ll be preparing press materials, please inform our press team as soon as possible -- no later than 48 hours after receiving the formal acceptance. Your manuscript will remain under strict press embargo until 2 pm Eastern Time on the date of publication. For more information, please contact onepress@plos.org.

Kind regards,

Jorddy Neves Cruz

Academic Editor

PLOS One

Additional Editor Comments (optional):

Reviewers' comments:

Reviewer's Responses to Questions

**Comments to the Author**

1. If the authors have adequately addressed your comments raised in a previous round of review and you feel that this manuscript is now acceptable for publication, you may indicate that here to bypass the “Comments to the Author” section, enter your conflict of interest statement in the “Confidential to Editor” section, and submit your "Accept" recommendation.

Reviewer #1: All comments have been addressed

**********

2. Is the manuscript technically sound, and do the data support the conclusions?

The manuscript must describe a technically sound piece of scientific research with data that supports the conclusions. Experiments must have been conducted rigorously, with appropriate controls, replication, and sample sizes. The conclusions must be drawn appropriately based on the data presented.

Reviewer #1: Yes

**********

3. Has the statistical analysis been performed appropriately and rigorously?

Reviewer #1: Yes

**********

4. Have the authors made all data underlying the findings in their manuscript fully available?

The PLOS Data policy requires authors to make all data underlying the findings described in their manuscript fully available without restriction, with rare exception (please refer to the Data Availability Statement in the manuscript PDF file). The data should be provided as part of the manuscript or its supporting information, or deposited to a public repository. For example, in addition to summary statistics, the data points behind means, medians and variance measures should be available. If there are restrictions on publicly sharing data—e.g. participant privacy or use of data from a third party—those must be specified.

Reviewer #1: Yes

**********

5. Is the manuscript presented in an intelligible fashion and written in standard English?

PLOS ONE does not copyedit accepted manuscripts, so the language in submitted articles must be clear, correct, and unambiguous. Any typographical or grammatical errors should be corrected at revision, so please note any specific errors here.

Reviewer #1: Yes

**********

6. Review Comments to the Author

Please use the space provided to explain your answers to the questions above. You may also include additional comments for the author, including concerns about dual publication, research ethics, or publication ethics. (Please upload your review as an attachment if it exceeds 20,000 characters)

Reviewer #1: The manuscript title "Biochemical and Antidiabetic Properties of Elaeocarpus angustifolius Blume: In vitro, In vivo, and In silico Insights" can be accepted in the present form.

**********

7. PLOS authors have the option to publish the peer review history of their article (what does this mean?). If published, this will include your full peer review and any attached files.

If you choose “no”, your identity will remain anonymous but your review may still be made public.

**Do you want your identity to be public for this peer review?** For information about this choice, including consent withdrawal, please see our Privacy Policy.

Reviewer #1: No

**********

# Editor report: Acceptance letter

**DOI:** 10.1371/journal.pone.0349796.r004

**Authors:** Jorddy Neves Cruz

PONE-D-25-60521R1

PLOS One

Dear Dr. Adhikari,

I'm pleased to inform you that your manuscript has been deemed suitable for publication in PLOS One. Congratulations! Your manuscript is now being handed over to our production team.

At this stage, our production department will prepare your paper for publication. This includes ensuring the following:

* All references, tables, and figures are properly cited

* All relevant supporting information is included in the manuscript submission,

* There are no issues that prevent the paper from being properly typeset

You will receive further instructions from the production team, including instructions on how to review your proof when it is ready. Please keep in mind that we are working through a large volume of accepted articles, so please give us a few days to review your paper and let you know the next and final steps.

Lastly, if your institution or institutions have a press office, please let them know about your upcoming paper now to help maximize its impact. If they'll be preparing press materials, please inform our press team within the next 48 hours. Your manuscript will remain under strict press embargo until 2 pm Eastern Time on the date of publication. For more information, please contact onepress@plos.org.

You will receive an invoice from PLOS for your publication fee after your manuscript has reached the completed accept phase. If you receive an email requesting payment before acceptance or for any other service, this may be a phishing scheme. Learn how to identify phishing emails and protect your accounts at https://explore.plos.org/phishing.

If we can help with anything else, please email us at customercare@plos.org.

Thank you for submitting your work to PLOS ONE and supporting open access.

Kind regards,

PLOS ONE Editorial Office Staff

on behalf of

Dr. Jorddy Neves Cruz

Academic Editor

PLOS One
